# Advances in mitophagy and mitochondrial apoptosis pathway-related drugs in glioblastoma treatment

**DOI:** 10.3389/fphar.2023.1211719

**Published:** 2023-06-30

**Authors:** Weiping Li, Xia Xu

**Affiliations:** Department of General Practice, Xiangya Hospital of Central South University, Changsha, Hunan, China

**Keywords:** drugs, glioblastoma, mitochondrial apoptosis, mitophagy, new developments

## Abstract

Glioblastoma (GBM) is the most common malignant tumor of the central nervous system (CNS). It is a leading cause of death among patients with intracranial malignant tumors. GBM exhibits intra- and inter-tumor heterogeneity, leading to drug resistance and eventual tumor recurrence. Conventional treatments for GBM include maximum surgical resection of glioma tissue, temozolomide administration, and radiotherapy, but these methods do not effectively halt cancer progression. Therefore, development of novel methods for the treatment of GBM and identification of new therapeutic targets are urgently required. In recent years, studies have shown that drugs related to mitophagy and mitochondrial apoptosis pathways can promote the death of glioblastoma cells by inducing mitochondrial damage, impairing adenosine triphosphate (ATP) synthesis, and depleting large amounts of ATP. Some studies have also shown that modern nano-drug delivery technology targeting mitochondria can achieve better drug release and deeper tissue penetration, suggesting that mitochondria could be a new target for intervention and therapy. The combination of drugs targeting mitochondrial apoptosis and autophagy pathways with nanotechnology is a promising novel approach for treating GBM.This article reviews the current status of drug therapy for GBM, drugs targeting mitophagy and mitochondrial apoptosis pathways, the potential of mitochondria as a new target for GBM treatment, the latest developments pertaining to GBM treatment, and promising directions for future research.

## 1 Introduction

Glioblastoma (GBM) is a malignant tumor that develops from astrocytes, which are cells that support nerve cells in the brain(Watson et al., 2023). It can also develop from mutations in specific pathways related to cell death and proliferation in different cells of the brain(Louis et al., 2021). Unfortunately, it has the lowest 5-year relative survival rate among central nervous system tumors (6.8%) (Ostrom et al., 2019). The first-line treatment for GBM includes maximal surgical resection followed by concomitant chemoradiotherapy and adjuvant chemotherapy (TMZ). (Szklener et al., 2022). After standard-of-care surgery and adjuvant chemotherapy, the approximate median survival is 14–16 months. It is mainly induced by its high resistance to radiotherapy and chemotherapy and the inability to remove the tumor tissue completely (Ohgaki and Kleihues, 2005; Lah et al., 2020). GBM-initiating cells (GICs), also known as GBM stem cells (GSCs), have the potential for self-renewal, multi-directional differentiation, and tumor initiation, which are associated with treatment resistance and relapse and are considered to be the cause of relapse in most patients with this devastating disease (He et al., 2021; Yi et al., 2019, Osuka and Van Meir, 2017). Temozolomide (TMZ) is a currently the first-line drug used for GBM treatment independent of the methylation state of O6-methylguanine methyltransferase(MGMT), which can induce DNA strand breaks during cell replication and thus promotes cell apoptosis(Hegi et al., 2019). Owing to the overexpression of MGMT and the lack of DNA repair pathways in GMB, TMZ-resistance is a major obstacle in improving the prognosis of patients with GBM (Chen et al., 2018; Lin et al., 2022a). Furthermore, phenotypic and genotypic heterogeneity (Banelli et al., 2017), hypoxic tumor environment (Ho et al., 2022), the presence of glioblastoma stem cells (Huang et al., 2020), abnormal signaling pathways (Yu et al., 2019; Liu et al., 2020a; Lee et al., 2022a), and notably, the existence of the blood–brain barrier (BBB) (Zou et al., 2022) result in a need for increased chemotherapeutic drug doses to reach effective concentrations of the drugs, which worsens the systemic side effects of the drugs (Oberoi et al., 2016). Therefore, further research, drug development, and identification of novel and effective drugs are urgently needed.

In recent years, natural products, synthetic drugs, and cytokines targeting the mitochondria have increasingly been applied for the prevention and treatment of various tumors, and their promising results in anti-tumor research and application are becoming evident. This review focuses on research progress into potential natural drug leads for inducing mitophagy or apoptotic pathways that may be relevant to GBM (Tab.1).

**TABLE 1:** Summary of main mitophagy and mitochondrial apoptosis pathway-related drugs in GBM treatment.

Classification	Drugs
Mitophagy pathway-related drugs	Silibinin
	Cannabidiol
	Gossypol (AT-101)
Apoptosis pathway-related drugs	Xanthohumol
	Pterostilbene
	Chrysophanol
	Shikonin
	Grape seeds
Mitophagy and mitochondrial apoptosis pathway-related drugs	Sinomenine

## 2 Mitophagy and GBM

### 2.1 Mitophagy

Mitochondria are important organelles that play important roles in cellular metabolism, including but not limited to the production of ATP via electron transport coupled with oxidative phosphorylation, tricarboxylic acid cycle, fatty acid β-oxidation, amino acid synthesis, calcium homeostasis, and iron metabolism (biosynthesis of heme and iron-sulfur clusters) (Zhang et al., 2022a). According to the International Cancer Genome Consortium and The Cancer Genome Atlas Program, mutations in mitochondrial DNA (mtDNA) can be detected in approximately 60% of solid tumors, and the accumulation of mutations in mtDNA can result in mitochondrial dysfunction(Leao et al., 2021). In glioma, mitochondrial function is impaired by marked alterations in the mitochondrial genome, resulting in altered morphology and abnormal bioenergetics, including increased ROS production(Lu and Ho, 2020). Mitochondrial dysfunction plays a crucial role in the regulation of several cancer intrinsic pathways related to tumor metabolism, survival, proliferation, and cell death in GBM (Lu and Ho, 2020).

Autophagy, morphologically characterized by the formation of autophagosomes or autolysosomes in the cytoplasm, is a degradation pathway through which intracellular materials or impaired organelles are transported to lysosomes for clearance (Levy et al., 2017). Autophagy has a dual function in GBM. As a tumor suppressor, it can destroy harmful unfolded proteins, oncogenic protein substrates, and damaged organelles (Batara et al., 2021). For instance, according to recent studies, breast cancer patients with brain metastases may benefit from therapeutic strategies aimed at targeting autophagy (Maiti and Hait, 2021).It may also have a role in protecting GBM cells by eliminating misfolded proteins generated during oxidative stress (Di Rita et al., 2018). Combining standard cancer treatment with the regulation of autophagy activity, by promoting or preventing autophagy using inducers or inhibitors based on tumorigenesis and cancer stages, has the potential to be a promising anti-cancer therapy (Li et al., 2020). Mitophagy refers to the selective removal of damaged mitochondria through the autophagy mechanism to maintain mitochondrial quality and rescue cells from death (Bravo-San et al., 2017; Wang et al., 2018).

**FIGURE 1:**
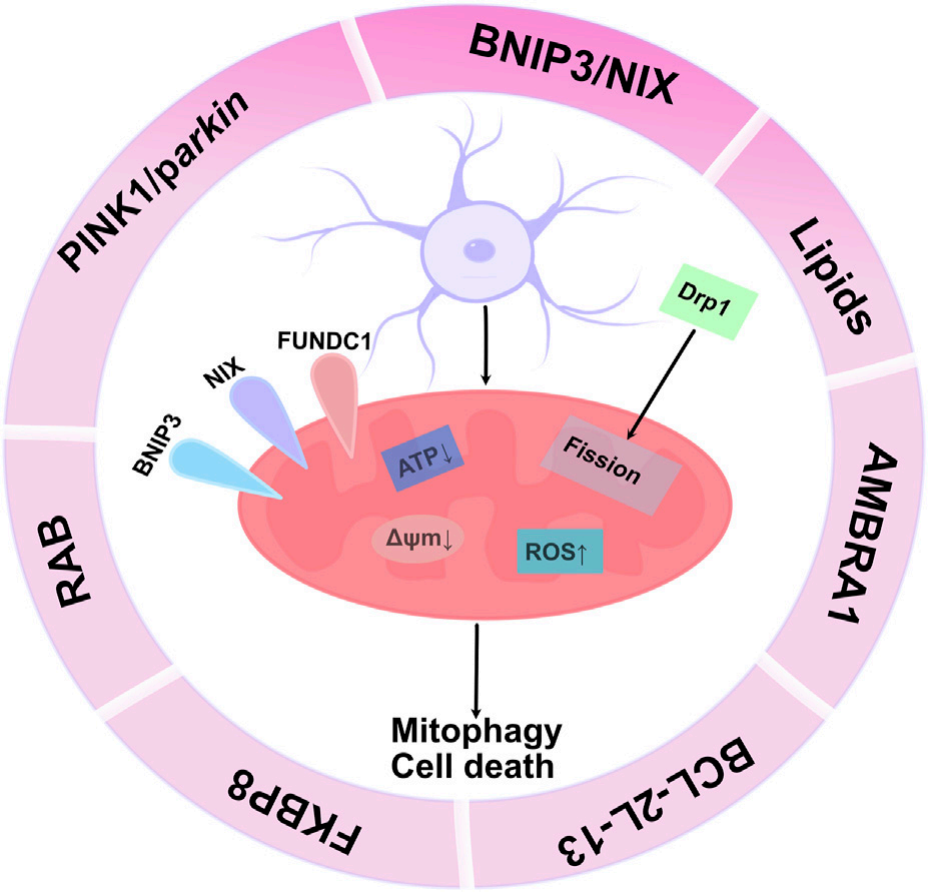
Molecular mechanism of mitophagy: The figure reflects mitophagy mediated by receptors (mainly BINP3, NIX, PINK1/parkin, FUNDC1, BCL-2L-13, lipids, RAB, FKBP8). These mitochondrial receptors mediate mitophagy by directly binding to LC3 on autophagosomes via a conserved LIR motif in their N-terminal region. Lipid accumulation on the mitochondrial outer membrane maintains cellular homeostasis, thereby regulating the mitophagy machinery. Hypoxia is an important stimulus that induces this process. PINK1/parkin-mediated mitophagy occurs in a ubiquitination-dependent manner, and ubiquitination of specific mitochondrial proteins enhances phosphorylation of ubiquitin on mitochondrial proteins by PINK1 to recruit mitophagy receptors and mediate the process of mitophagy. After further polyubiquitination, parkin recruits adapter proteins (such as p62/SQSTM1, OPTN) and interacts with LC3 on the membrane surface of autophagosomes to promote mitophagy.

These pathways can be classified into typical and atypical. The typical pathway mainly includes PINK1/parkin-, BNIP3/NIX-, and FUNDC1-mediated mitophagy, whereas the atypical pathway mainly includes lipid-, AMBRA1-, BCL2L13-, FKBP8-, and RAB-mediated mitophagy (Vara-Perez et al., 2019). Of note, the autophagy/lysosomal pathway that removes damaged mitochondria (i.e., mitophagy) is impaired in patients with Alzheimer’s disease, which leads to the accumulation of dysfunctional mitochondria, leading to synaptic dysfunction and cognitive deficits (Kerr et al., 2017). Dopaminergic neurons selectively fail to execute mitophagy, which promotes their survival(Bernardini et al., 2017; Katayama et al., 2020) within lesions in a mouse model of Parkinson’s disease. Rapamycin reduces cisplatin-mediated nephrotoxicity by stimulating PINK1/parkin-mediated mitophagy in renal tubular cells, reducing tissue damage caused by chemotherapy (Wang et al., 2018). Accordingly, mitophagy plays a crucial role in maintaining cellular homeostasis and is a major pathway for the degradation of dysfunctional or damaged mitochondria. Moreover, mitophagy is also a programmed event involved in developmental and differentiation processes, including the elimination of paternal mitochondria from fertilized eggs (Song et al., 2021), as well as the removal of mitochondria during erythropoiesis and muscle differentiation (Senft and Ronai, 2016; Panigrahi et al., 2020) (Figure 1).

### 2.2 Relationship between mitophagy and GBM

Activation of mitophagy has been used in the treatment of GBM(Zhou et al., 2020; Wang et al., 2022; Cammarata et al., 2023).It can relieve stress and suppressing tumors by eliminating dysfunctional mitochondria, and mitophagy-mediated clearance of pro-apoptotic mitochondria may provide cytoprotective benefits(Panigrahi et al., 2020). In recent years, studies have shown that drugs related to mitophagy pathways can promote the death of GBM cells by inducing mitochondrial damage, impairing ATP synthesis, and depleting ATP in large quantities. The induction of lethal autophagy has become a strategy to eliminate GBM cells, which is reportedly an effective method to eradicate cancer cells(Maiti et al., 2019; Meng et al., 2022; Rademaker et al., 2022).

### 2.3 Drugs related to mitophagy

#### 2.3.1 Silibinin

Silibinin is a flavonoid extracted and isolated from the fruit of the chrysanthemum plant Silybum marianum (Tuli et al., 2021). It has been widely used for the treatment and prevention of various hepatobiliary disorders, including alcoholic liver disease, non-alcoholic fatty liver disease, and mushroom poisoning (Abenavoli et al., 2018; Hosseinabadi et al., 2019; Wang et al., 2020a). Recent studies have demonstrated the broad-spectrum anti-cancer effects of silibinin against most types of cancer cells(Jahanafrooz et al., 2018). For example, it can inhibit the migration and invasion of breast cancer MDA-MB-231 cells through induction of mitochondrial fusion(Si et al., 2020). In hepatocellular carcinoma, silibinin has been found to effectively abate hepatocarcinogenesis and hepatocellular carcinoma growth by regulating various signaling pathways including HGF/c-Met, Wnt/β-catenin and PI3K/Akt/mTOR(Yassin et al., 2022). In cholangiocarcinoma, silibinin has the ability to inhibit cholangiocarcinoma through the ERK/mitochondrial apoptotic pathway, which makes silibinin a potential anti-tumor drug candidate for cholangiocarcinoma treatment(Bai et al., 2022).

Considering that silibinin has extremely high antioxidant and anti-tumor properties, it has drawn our attention to its potential use in the treatment of GBM.BNIP3, a member of the Bcl-2 family of pro-apoptotic proteins and a receptor for mitophagy, exhibits context-dependent roles in cancer(Gorbunova et al., 2020; Gorbunova et al., 2020; Vara-Perez et al., 2021).It targets mitochondria and could induce mitochondrial damage and nuclear translocation of AIF6 (Su et al., 2016). A study using GBM cell lines and nude mice with xenografted GBM has confirmed that silibinin could induce mitophagy in GBM, and that autophagy can promote silibinin-induced BNIP3 overexpression and its accumulation in the mitochondria, thereby triggering AIF-dependent death in GBM cells (Wang et al., 2020b). Moreover, silibinin has also been shown to inhibit GBM cell migration by inhibiting MMP-2 and -9 and improving TMZ-resistance in GBM cells (Zhai et al., 2021; Wong et al., 2023). Silibinin have potential uses for patients with GBM. However, like other polyphenols, faces the challenge of low bioavailability, which impedes its potential as a transformative chemotherapeutic drug(Tuli et al., 2021). At the same time, further clinical research is also needed to better understand the potential toxicity and risks associated with the drug’s use in treating GBM. This will provide more reliable evidence to support clinical treatment of GBM.

#### 2.3.2 Cannabidiol

Cannabidiol (CBD), the main active component of medical cannabis, is extracted from the wild hemp (Karimi-Haghighi et al., 2022). It easily passes through the BBB, is highly safe, and has anti-proliferation and anti-invasion activities against various cancers (Valenti et al., 2022; Ammendolia et al., 2023). The literature indicates that in many animal cancer models, CBD has shown potential in inhibiting the progression of various types of cancers, including in GBM, breast(Kiskova et al., 2019; Valenti et al., 2022), lung(Milian et al., 2022; Misri et al., 2022), prostate(Mahmoud et al., 2023), colon cancer(Jeong et al., 2019; Lee et al., 2022b; Yuksel et al., 2023), and melanoma(Bachari et al., 2020). CBD has emerged as a promising agent in the treatment of glioma cells due to its ability to inhibit their proliferation and promote cell death. This effect is mainly achieved by targeting the mitophagy pathway, which has gained significant attention in recent research.

Transient receptor potential vanilloid 4 (TRPV4) is a widely expressed multimodal-gated ion channel that plays a pivotal role in many physiological and pathophysiological processes (Grace et al., 2017; Muller and Reggio, 2020). Its expression in human brain basement membrane tissue is closely related to tumor grade and prognosis (Yang et al., 2020). CBD can induce mitophagy by activating endoplasmic reticulum stress via the TRPV4–ATF4–DDIT3–TRIB3–AKT–MTOR axis. TRPV4 expression in human GBM tissues correlates with both tumor grade and poor survival, suggesting that TRPV4 could be an attractive therapeutic target and biomarker for GBM (Huang et al., 2021a). CBD can also lead to abnormal stability of the plasma membrane by affecting the homeostasis of GBM lipid metabolism, thereby promoting the phagocytosis of tumor cells by macrophages and exerting an anti-GBM effect (Khodadadi et al., 2021; Genovese et al., 2022). These two mechanisms synergistically inhibit the formation and development of GBM, indicating that CBD has great clinical application prospects as an anti-GBM medicine et al., 2021).

Simultaneously, compared with single drug treatment alone, the combined treatment of CBD and TMZ more effectively targeted GBM patients, significantly inhibiting the growth of GBM cells and prolonging survival time, suggesting that CBD can effectively enhance the anti-tumor effect of TMZ in GBM (Lopez-Valero et al., 2018; Huang et al., 2021b). Furthermore, in the first study on the CBD-induced anti-tumor effects of RELA Ser311 phosphorylation, ROS was shown to serve as a biomarker for stratifying patients who may benefit from CBD treatment (Volmar et al., 2021).

#### 2.3.3 Gossypol (AT-101)

Gossypol (2,2ʹ-bis-(formyl-1,6,7-trihydroxy-5-isopropyl-3-methylnaphthalene), a BH3-mimetic compound naturally present in cottonseed, exerts anti-tumor effects by targeting various signal transduction pathways. It has been extensively studied in clinical trials, where it has shown good tolerability and safety (Benvenuto et al., 2018; Yurekli et al., 2018). However, recent studies have found that it is the (-)-enantiomer of gossypol, namely (-)-gossypol (also known as AT-101), rather than (+)-gossypol or racemic gossypol, that has significant anti-cancer properties (Benvenuto et al., 2018). Therefore, the development of single-isomer pharmaceutical preparations can avoid potential adverse reactions. Thus far, AT-101 has been considered a promising anti-cancer drug for the treatment of various tumors, including multiple myeloma (Ailawadhi et al., 2023), adrenal cortical carcinoma (Yurekli et al., 2018), esophagus cancer (Que et al., 2019), breast cancer (Bulut et al., 2020), lung cancer (Ahmad et al., 2021; Renner et al., 2022), and prostate cancer (Aktepe and Yukselten, 2022).

HMOX1 is an inducible enzyme that catalyzes the degradation of oxidized preheme and is also involved in mitochondrial biogenesis and mitophagy (Constantin et al., 2012; Hull et al., 2016). AT-101 can promote GBM cell death by inducing overactivation of HMOX1 and the autophagy receptors BNIP3 and BNIP3L, causing early mitochondrial dysfunction and marked loss of mitochondrial mass/protein (Meyer et al., 2018). It also suppresses the growth of TMZ-resistant glioblastoma (Kim et al., 2019). Mitochondrial respiration and mitochondrial permeability transition pore opening were impaired after AT-101 treatment, suggesting that mitochondrial dysfunction is a key driver of AT-101-induced cell demise (Meyer et al., 2018). Because the AT-101 molecule is hydrophobic, oral administration greatly reduces its bioavailability, and gastrointestinal side effects can easily be caused. Therefore, the cyclic RGD (cRGD)-decorated mixed liposome (cRGD-LP) nanopreparation for the tumor-targeted delivery of AT-101 (abbreviated as Gos hereafter) came into being (Xie et al., 2019a; Liu et al., 2022). This nanoformulation enhanced tumor engraftment *in vivo*, possibly due to cRGD binding to the αvβ3 integrin on tumors and tumor cells, enhancing tumor targeting (Liu et al., 2022). Moreover, some studies have also shown that arsenic trioxide-mediated hedgehog/notch inhibition can interfere with DNA double-stranded break repair by reducing the expression of CHEK1 and CHEK2, synergistically targeting GSC along with AT-101 (Linder et al., 2019). AT-101 combined with demethoxycurcumin can enhance the inhibitory effect on the proliferation of glioblastoma cells (Mehner et al., 2020), suggesting that combination therapy with different agents may be an option to overcome drug resistance in GBM cells effectively, in a long-term treatment strategy.

## 3 Mitochondrial apoptosis and GBM

### 3.1 Mitochondrial apoptosis

**FIGURE 2:**
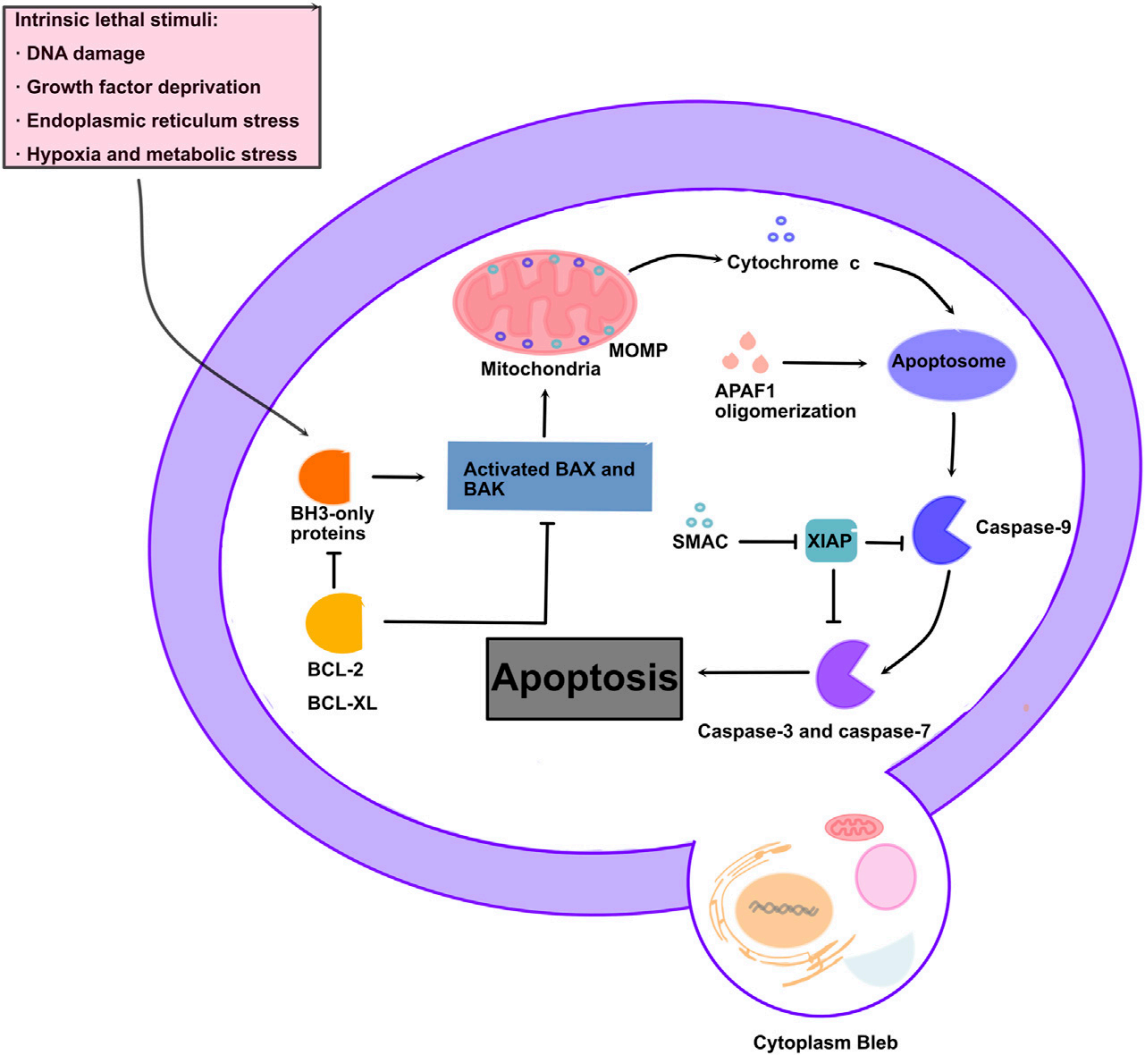
Molecular mechanism of mitochondrial apoptosis: Apoptotic stresses promote accumulation of BH3-only proteins leading to BAX/BAK oligomerization, MOMP, and release of intermembrane space proteins. Cytochrome c leads to apoptosome formation, which results in caspase activation and apoptosis.

Mitochondria serve as vital organelles in diverse cellular functions, including oxidative phosphorylation, ROS, and calcium signaling, as well as intermediate metabolite synthesis required for cell growth and motility(Bhargava and Schnellmann, 2017). ROS are a crucial class of molecules directly involved in the regulation of mitochondrial function, mainly produced by mitochondrial oxidative phosphorylation. Various cellular metabolic processes are associated with ROS, including transcription factor activation, gene expression, and cell differentiation and proliferation (Thannickal and Fanburg, 2000). Apoptosis is a type of programmed cell death that maintains the homeostasis of the internal environment, which is mainly regulated by the activation of the caspase cascade (Zimmermann et al., 2001). Caspase-3 is considered as the most important regulator of apoptosis, while caspase-9 is considered to be the master regulator of mitochondria-mediated apoptosis (Batoon et al., 2023; Cao et al., 2023). Apoptosis is controlled by intrinsic (mitochondrial pathway) and extrinsic pathways, and the intrinsic pathway is regulated by the BCL-2 family, including the anti-apoptotic activator BCL-xL and proapoptotic effector BAX(Lindenboim et al., 2000). Cell stress induces the proapoptotic effector BAX to induce cell apoptosis by inducing the release of cytochrome-c (Cyt-C), a key component of the mitochondrial electron transport chain, into the cytoplasm (Finucane et al., 1999; Desagher and Martinou, 2000). In the extrinsic pathway, caspase-8 cleaves and activates procaspase-3 (Boatright and Salvesen, 2003). However, the result of both pathways is caspase activation and the cleavage of specific cellular substrates, leading to morphological and biochemical changes associated with an apoptotic phenotype (Lee et al., 2020). In this process, apoptosis is characterized by the formation of apoptotic bodies, containing the contents of dead cells, which will be engulfed by the surrounding cells without causing content leakage or damage to the surrounding cells (Li et al., 2021) (Figure 2).

### 3.2 Relationship between mitochondrial apoptosis and GBM

The accumulation of intracellular ROS can cause carcinogenesis. In GBM cells that require high levels of ROS, if ROS are lower than the minimum level for GBM cell survival, it may induce intracellular signaling disturbances and apoptosis (Huang et al., 2021a; Huangfu et al., 2021). The accumulation of mutations in mitochondrial DNA (mtDNA) contributes to mitochondrial dysfunction, which plays a crucial role in the pathogenesis of GBM. This dysfunction leads to abnormal energy and reactive oxygen species production, as well as resistance to apoptosis and chemotherapeutic agents(Leao et al., 2021).While many chemotherapeutic drugs play a tumor-killing role by inducing ROS and enhancing oxidative stress, they can also damage the mitochondria and DNA of normal cells and even induce carcinogenesis in other cells (Kleih et al., 2019). Therefore, regulating the level of ROS in tumor and normal tissues and selectively killing tumor cells has great clinical significance (Di Meo et al., 2022). Mitochondria are considered to be novel targets for cancer intervention and therapy (Xu et al., 2009). It can induce apoptosis in GBM cells by disrupting the balance in the anti-oxidant system, which are important mechanisms in the research of anti-tumor therapies (Benlloch et al., 2016; Feng et al., 2016).

### 3.3 Drugs related to mitochondrial apoptosis

#### 3.3.1 Xanthohumol

Xanthohumol (XN), a natural compound found in hops, is an isoprene flavonoid with a wide range of biological activities, including anti-inflammatory, anti-oxidant, anti-cancer, antibacterial, and lipid lowering (Lin et al., 2022c; Neumann et al., 2022). Since flavonoids readily cross the BBB *in vivo*, they are considered potential drug leads for treating disease. Several studies have shown that XN has anti-GBM effects. It can not only inhibit the IGFBP2/AKT/BCL-2 pathway and activate the P53 signaling pathway to participate in XN-induced GBM cell apoptosis (Chen et al., 2016), but it also induces apoptosis of glial pathway cells by increasing ROS and activating MAPK pathways (Festa et al., 2011). Hou et al. confirmed that XN can inhibit C6 proliferation, trigger mitochondrial stress, and induce cell death in a concentration- and time-dependent manner (Hou et al., 2021). Following treatment of GBM cells with XN, the cell cycle was blocked at the G0/G1 phase, and XN induced AIF-mediated apoptosis, which was accompanied by mitochondrial structure and function impairment, as well as mitophagy blockage (Hou et al., 2021). In contrast, mitochondrial injury not only disrupts ATP synthesis in cells but also consumed large amounts of ATP to maintain intracellular stability. This vicious cycle exacerbates cellular energy consumption. The DNA repair machinery is a tool to remove DNA damage for the maintenance of genomic integrity in normal cells and paradoxically plays a crucial role in driving the development of drug resistance and tumor recurrence (Huang and Zhou, 2021). The results of Ho et al. showed that XN could enhance the cytotoxicity of TMZ by inhibiting the DNA repair system and could be used as an adjuvant drug in the treatment of patients with GBM with DNA repair activation (Ho et al., 2020). Moreover, XN also reduces the invasiveness of GBM cells by inhibiting the signaling of stromal interacting molecule 1 (STIM1), indicating that XN may be a good GBM therapeutic agent (Ho et al., 2018). Elucidating the XN-mediated molecular mechanism may provide novel strategies for future drug development and tumor research.

#### 3.3.2 Pterostilbene

As a methylated derivative of resveratrol, pterostilbene (PTE) has higher biological activity and safety than resveratrol and is mainly found in blueberries and grapes (Rimando et al., 2004; Ruiz et al., 2009; Chang et al., 2012). PTE has a wide range of biological functions, including anti-tumor, anti-oxidation, anti-inflammatory, apoptosis, cardiovascular protection, anti-proliferation, and antibacterial activities (Chen et al., 2020), gallbladder (Tong et al., 2021), breast (Harandi-Zadeh et al., 2021; Kumar et al., 2021), colon (Wawszczyk et al., 2022), cervical (Shin et al., 2020), prostate (Hemani et al., 2022), and lung cancers (Bracht et al., 2019). In GBM, PTE can induce the loss of mitochondrial membrane potential and production of reactive oxygen species (ROS) (Gao et al., 2021) and activate the FAS/FASL pathway and caspase-3, thereby inhibiting proliferation and inducing GBM cell apoptosis (Tan et al., 2019; Gao et al., 2021). Moreover, given that PTE presents highly bioavailability and easily crosses the BBB, PTE administration can serve as a novel treatment for patients with GBM (Ma et al., 2019). Based on the abovementioned experimental results, PTE has a high research value and development prospects in the field of GBM drug treatment.

#### 3.3.3 Chrysophanol

Chrysophanol (1, 8-dihydroxy-3-methyl-9, 10-anthraquinone) is a phytochemical extracted from Rheum officinale (rhubarb), which has been utilized as a traditional Chinese herbal medicine (Yusuf et al., 2019; Su et al., 2020). It has various pharmacological effects, including anti-cancer, antioxidant, neuroprotective, antibacterial, antiviral, and blood lipid-regulation effects. Studies have shown that chrysophanol can attenuate hepatic stellate cell-induced endoplasmic reticulum fibrosis by regulating hepatitis B virus stress and iron concentration (Kuo et al., 2020). Moreover, it can inhibit the growth and metastasis of T-cell acute lymphoblastic leukemia through the miR-9/PD-L1 axis (Yin et al., 2021), regulating the effect of the microRNA-27b-3p/peroxisome proliferator-activated receptor γ axis on sepsis-induced acute myocardium damage protection (Park et al., 2022).

Moreover, the application of chrysophanol for cancer treatment is also increasing. For instance, chrysophanol promotes cell morphological changes, induces cell apoptosis through DNA damage, and arrests S phase cell cycle among patients with liver cancer (Ni et al., 2012). In patients with lung cancer, chrysophanol expresses anti-cancer activity by regulating the ROS/HIF-1a/VEGF signaling pathway (Zhang et al., 2020a; Zhang et al., 2021a). In patients with GBM, it has been discovered that chrysophanol increased the accumulation of ROS in the mitochondria of GBM cells, promoting the release of Cyt-C from the mitochondria to the cytoplasm and, thereby, causing GBM cell apoptosis (Gu et al., 2021). Chrysophanol regulates the anti-cancer effect on GBM cells by activating the mitochondrial apoptosis pathway, indicating that it may serve as an innovative chemotherapeutic agent for GBM. However, chrysophanol has obvious hepatotoxicity and nephrotoxicity. Nevertheless, pharmacokinetics has shown that chrysophanol combined with other drugs can reduce toxicity and improve efficacy (Xie et al., 2019b).

#### 3.3.4 Shikonin

Shikonin is the main bioactive component extracted from the root of Lithospermum erythrorhizon, which has various bioactivities related to cancer treatment, inflammation, and wound healing. Many studies have shown that shikonin has strong anti-cancer effects on leukemia, gastrointestinal cancer, pancreatic cancer, lung cancer, breast cancer, and urogenital organ cancer, by inhibiting cell proliferation and migration, and inducing apoptosis and necroptosis (Guo et al., 2019). A clinical trial conducted by Guo et al. reported that, among 19 patients suffering from late-stage lung cancer who were not subjected to surgery, chemotherapy, or radiotherapy, the tumor diameter decreased by more than 25% after treatment with shikonin, posing a remission rate of 37% and a 1-year survival rate of 47% (Boulos et al., 2019). Shikonin is a potent inducer of necrotizing apoptosis in cancer cells. In terms of pharmacological mechanism, anti-glioma effect of shikonin by interfering with endoplasmic reticulum stress-mediated tumor apoptosis targeting Caspase-3, and Bax/Bak-induced mitochondrial outer membrane permeabilization (MOMP) triggering cancer cell apoptosis (Ma et al., 2020). ROS is the executor of necrotizing apoptosis. Shikonin increases intracellular ROS levels by targeting both NOX1 and the mitochondrial respiratory chain complex (Yang et al., 2014). RIP1 and RIP3 can modulate shikonin-induced ROS overproduction by targeting the mitochondria and promoting RIP1/RIP3-dependent necroptosis in GBM cells (Lu et al., 2017). Shikonin has shown great promise as a potential drug for treating glioma by targeting the mitochondrial apoptosis pathway. In order to achieve greater precision and efficacy in treating glioma, it is necessary to consider the shikonin’s ability to cross the blood-brain barrier. Wang et al. developed an AS1411 aptamer/hyaluronic acid-bifunctionalized microemulsion co-loading shikonin and docetaxel (AS1411/SKN&DTX-M), which has the ability to penetrate the BBB according to their research report. The codelivery of shikonin and docetaxel through bifunctionalization with hyaluronic acid and AS1411 aptamer presents a promising approach for anti-GBM therapy using dual-drug therapy (Wang et al., 2019).

#### 3.3.5 Grape seeds

Grape seeds are the seeds of Vitis vinifera. Grape seed proanthocyanidins (GSP) is a general term for a large class of polyphenolic compounds that have antioxidant activity. GSP has various biological activities and has been proven to have good anti-tumor effects, as well as certain inhibitory effects on cervical cancer (Li et al., 2022a), carcinoma of the urinary bladder (Yang et al., 2021a), lung cancer (Xu et al., 2021a; Zhang et al., 2021; Mao et al., 2023), colon cancer (Aiello et al., 2019; Zhang et al., 2019), liver cancer (Feng et al., 2019), prostate cancer (Chen and Yu, 2019), among others. In a study on liver cancer cells, GSP was found to trigger ROS production, decrease matrix-metalloproteinases (MMPs), and increase caspase-3 activity in HepG2 cells (Wang et al., 2020), proving that GSPs may induce ROS production and, consequently, lead to MMP reduction and caspase-3 activation. This ultimately induces HepG2 cell apoptosis. GSP can reverse EMT by inhibiting the TGF-β signaling pathway, effectively inhibiting the migration and invasion of bladder cancer (BC) cells (Yang et al., 2021b), suggesting that GSP can be used as a potential chemotherapy drug for BC. GSP can also reduce the proliferation activity of cancer cells (Habib et al., 2022). The mechanism of GSP pertaining to GBM is related to the inhibition of proliferation, induction of apoptosis, arrest of the cell cycle, and inhibition of angiogenesis and metastasis (Yang et al., 2021b). Grape seed, as a natural anti-cancer drug, holds great promise for the treatment of glioma. However, further clinical research is necessary to fully elucidate its role in the treatment mechanism.

### 3.4 Drugs related to mitochondrial apoptosis and mitophagy

#### 3.4.1 Sinomenine

The alkaloid sinomenine (SIN), namely 7,8-didehydro-4-hydroxy-3,7-dimethoxy-17-methylmorphinan-6-one (C19H23NO4), is extracted from the rhizome of the traditional Chinese medicine plant Sinomenium acutum (Zheng et al., 2021). SIN has anti-inflammatory effects and has been used to treat rheumatoid diseases in humans (Lin et al., 2022b; Chen et al., 2022). In recent years, SIN and its derivatives have been reported to have strong anti-tumor activity against various tumors, including BC (Xu et al., 2021b), prostate (Xu et al., 2019), papillary thyroid (Zhang et al., 2022b), breast (Li et al., 2022b; Gao et al., 2022), ovarian (Qu et al., 2021), and lung cancers (Bai et al., 2021). SIN can inhibit cell proliferation (Sun et al., 2018a; He et al., 2018), induce apoptosis (Liu et al., 2019) and arrest the cell cycle at the G0/G1 phase in various cancers (Yang et al., 2021a). SINI-WCJ-33 (SW33, C33H51NO5), a SIN-derivative obtained by the acylation of 4-hydroxyl and 14-carboxylic acid, can inhibit the proliferation, migration, invasion, and colony formation of human glioblastoma cell lines (Zheng et al., 2021). This derivative has higher anti-GBM activity and safety than its parent compound (Liu et al., 2019). The CCNB1/CDC2 complex is a key mediator of the G2/M checkpoint (Park et al., 2000; Taylor and Stark, 2001; Cheng et al., 2016). The polo-like kinase (PLK1)-dependent phosphorylation of CDC25C is required for normal cell cycle progression from the G2/M phase (Liu et al., 2020b; Tang et al., 2020). SW33 can reduce the expression of P-CDC2, CDC2, and CCNB1, as well as the protein levels of P-PLK1 and PCDC25C in GBM cells. It can also increase the expression of P53 and its transcriptional target P21, finally leading to the arrest of the GBM cell cycle in the G2/M phase, causing mitochondrial dysfunction, consequently releasing Cyt-C, activating caspase 3/9, and inducing mitochondrial apoptosis (Zheng et al., 2021).

In addition, PI3K/AKT/MTOR, MAPK/MTOR, and AMPK/MTOR have been widely reported to activate mitophagy (Zhang et al., 2020b; Liu et al., 2021). Zheng et al. have shown that SW33 can induce autophagy through the PI3K/AKT/MTOR and AMPK/MTOR signaling pathways in patients with GBM, thus playing an anti-GBM role, significantly inhibiting tumorigenesis, without having obvious adverse effects on the body (Zheng et al., 2021). Taken together, all these results suggest that SW33 may be a promising drug for the treatment of GBM.

## 4 New advances in drug therapy for GBM

### 4.1 The application of nanotechnology in GBM

The BBB comprises multiple components with barrier functions, including polarized endothelial cells connected by continuous adhesive and tight junctions, endothelial and parenchymal basement membranes, pericytes, and astrocyte foot processes (endfeet) (Steeg, 2021). As a barrier between circulating blood and brain parenchyma, it can prevent blood-borne pathogens or toxic substances from entering the CNS, maintain the dynamic balance of the CNS, and prevent the effective passage of cancer treatment drugs, including antibodies and miRNAs (Sarkaria et al., 2018). The concept of the BBB was first proposed by Edwin Goldman in 1913, who observed the limited transport of dye between the blood and brain. After injecting dye into the veins and CSF of animals, dye was distributed in almost all organs, except the brain (Langen et al., 2019). The disruption of the BBB during tumor progression results in the formation of the blood-tumor barrier (BTB) (Steeg, 2021). While the BTB is more permeable than the BBB, its uneven permeability to molecules of different sizes and uneven blood flow can lead to less than ideal drug accumulation in brain tumors(Arvanitis et al., 2020; Steeg, 2021).With significant advances in nanotechnology, various inorganic/organic/natural nanomaterials that target ligands and/or cell-penetrating peptide (CPP) surface modifications through the BBB have been created to help drugs cross the BBB to induce mitochondrial dysfunction for highly precise therapy (Tang et al., 2019).

#### 4.1.1 Resveratrol

Resveratrol (3,5,4′-trihydroxystilbene) (RES) is a naturally occurring polyphenol and phytoalexin that is abundant in red wine, berries, peanuts, and soybeans and has anti-inflammatory, anti-oxidant, anti-cancer, cardioprotective, and neuroprotective effects (Baur and Sinclair, 2006; Catalgol et al., 2012; Neves et al., 2012). Resveratrol is effective in the treatment of GBM through various mechanisms, but its bioavailability is severely reduced due to its poor water solubility, short biological half-life (approximately 9–14 min for primary molecules), chemical instability (oxidation and photosensitivity), and rapid metabolism and elimination (Jhaveri et al., 2018). If its shortcomings as a free drug can be overcome, its *in vitro* activity could be enhanced, and the relevant therapeutic effect could be improved. Triphenylphosphine (TPP+) is a lipophilic cation that can couple many bioactive molecules to achieve mitochondrial targeting (Wang et al., 2021). According to a report, paclitaxel-loaded liposomes prepared using TPP- modified polyethylene glycol-phosphatidylethanolamine (PEGPE) have been shown to be effective in targeting mitochondria in cancer cells (Biswas et al., 2012). Loading RES into PEGylated liposomes (RES-Ls) has been reported to overcome its drawbacks as a free drug (Fu et al., 2021). Furthermore, transferrin is overexpressed on most cancer cells, and transferrin-targeted RES-Ls may be an effective nanomedicine for the treatment of various cancers, including GBM, even though their biodistribution *in vivo* and ability to cross the BBB remain unknown.

#### 4.1.2 Berberine

Berberine (BBR) is a natural compound isolated from Chinese herbal medicine, including the Coptis root (Huang Lian) and Amur corktree (Huang Bai). It has a wide range of pharmacological effects, including antidiarrheal, antibacterial, antioxidant, anti-inflammatory, and anti-tumor aspects (Li-Weber, 2013; Li et al., 2015). BBR can inhibit GBM cell growth, reduce cellular viability, and induce oncosis-like death (cell swelling, cytoplasmic vacuoles, and plasma membrane blebbing) (Sun et al., 2018b). We also found that BBR induces autophagy as a protective effect and decreases the oxygen consumption rate, which could inhibit mitochondrial aerobic respiration by repressing phosphorylated extracellular regulated protein kinases (p-ERK1/2), reducing its energy production efficiency and, thereby, reducing metabolic activity (Sun et al., 2018b). The most challenging aspect related to BBR or other therapeutics in GBM is crossing the BBB. Glucose-coated nanodrugs and fructose-coated nanoparticles can provide 10–100-times more uptake by tumor cells in various models (Hu et al., 2015). The formation of nanoshapes by simply dissolving BBR into 5% glucose solution provides a promising strategy for drugs to cross the BBB (Wang et al., 2020).

### 4.2 Sonodynamic therapy

Sonodynamic therapy (SDT) is a technique that involves using focused ultrasound (FUS) to increase the sensitivity of tumors to sonosensitizers during sonication (Mess et al., 2023).It has shown promise as a cancer therapeutic modality for GBM due to its high tissue penetration and minimal radiation damage to normal tissues (Zhang et al., 2021c). Despite the potential of SDT in eliminating tumor cells, its effectiveness is limited by the BBB and the low accumulation rate of sonosensitizers (Guo et al., 2022). As a result, complete eradication of tumor cells cannot be guaranteed through SDT.Therefore, to improve the efficiency of drug delivery and further reduce adverse reactions, ultrasound-targeted microbubble destruction has been developed. It is a non-invasive technology that combines low-intensity FUS and microbubbles (MBs), which can transiently and reversibly destroy the BBB and promote drug delivery in the brain with a high degree of spatial and temporal specificity (Gorick et al., 2018). Low-intensity FUS has been explored as a drug delivery platform for the treatment of brain diseases (Landhuis, 2017), which can promote the deep penetration of SDT and the accumulation of tumor-specific sonosensitizing agents (Yeshurun and Azhari, 2016). SDT often concomitantly initiates an autophagic response during tumor cell apoptosis induction (Zhao et al., 2011). Excessive ROS production by ACL-SDT induces mitochondrial dysfunction and leads to MAPK/p38-PINK1-PRKN-dependent mitophagy (Qu et al., 2020). Mitophagy plays a protective role under oxidative stress, and inhibition of the degradation pathway significantly enhances the SDT-induced apoptosis of GBM cells (Qu et al., 2020). The lysosomal chemoattractor hydroxychloroquine (HCQ) is the only clinically available autophagy inhibitor (Cook et al., 2014). Qu et al. designed an “all-in-one” nanosensitization platform incorporating Ce6 and HCQ into angiopeptide-2 peptide-modified liposomes and designated a smart nanosensitizer, that can be used to treat GBMs *in situ* (Qu et al., 2020). Combining autophagy inhibitors with non-invasive SDT therapy provides a promising anti-GBM strategy, and the “all-in-one” nanosensitization platform is expected to be extended to other sonotheranostics in future.Besides, the efficiency of SDT can be enhanced by using a nano-platform biodegradation technology called CSI. This involves encapsulating catalase (CAT) into silica nanoparticles (CAT@SiO2) to alleviate tumor hypoxia, and then loading it with the sonosensitizer indocyanine green, which significantly improves the efficacy of SDT(Wu et al., 2022). The combination of SDT and natural drugs targeted to mitochondria can significantly enhance the therapeutic efficacy against glioma, which holds great importance for precise treatment of this disease.

## 5 Summary

GBM is the most common primary malignant brain tumor with high metabolic activity. Currently, GBM is treated by removing the tumor to the maximum extent and combining it with chemotherapy (Molinaro et al., 2022). However, due to its invasiveness, the total resection rate is low, the residual tumor tissue has obvious resistance to radiotherapy and chemotherapy, and the long-term survival rate of patients with GBM is low (Yi et al., 2019). The presence of the BBB further complicates the treatment process. Despite significant progress in the standard of care for GBM, including surgery, radiation therapy, and medical therapy such as chemotherapy with TMZ, patient outcomes remain extremely poor with a low median overall survival rate. GBM is still considered a fatal disease with limited treatment options.Given the extremely low survival rates of currently approved treatments for GBM, new therapeutic strategies are urgently needed. The clinical reality of the BBB contribution to GBM treatment failure suggests that renewed efforts to optimize BBB disruption techniques, develop BBB penetrators, and perfect impenetrable drug delivery technologies that bypass the BBB are the focus of current GBM treatment research. With the development of comprehensive treatment for glioblastoma in recent years, the anti-cancer effects of natural products and phytochemicals commonly used in traditional Chinese medicine continue to attract widespread attention. But the BBB presents a challenge for the effective delivery of anticancer drugs to the brain, limiting their curative effects.Modern nano-drug delivery technology targeting mitochondria can achieve better drug release and deeper tissue penetration, suggesting that mitochondria could be a new target for intervention and therapy. The combination of drug targeting mitochondrial apoptosis and autophagy pathways with nanotechnology is a promising novel approach for treating GBM. However, it is a particularly challenging task to engineer nanoformulations that can perfectly target mitochondrial abnormalities in tumor cells without causing toxic effects on nearby normal cells. Since most of our experiments were carried out on animal models, further research is needed to explore the safety parameters of ultrasound in GBM.With the rapid advances in knowledge and nanomedicine for GBM, increasing numbers of molecular targets have been identified, providing a solid foundation for the development of precise nanotherapeutic systems in future.We look forward to the development of more effective drugs for GBM treatment, focused on the mitochondrial pathway, and the emergence of more mature nanoagents combined with nanotechnology to kill tumor cells specifically, improving the therapeutic effects of medicine for GBM.

## References

[B1] Abenavoli L. Izzo A. A. Milic N. Cicala C. Santini A. Capasso R. (2018). Milk thistle (Silybum marianum): A concise overview on its chemistry, pharmacological, and nutraceutical uses in liver diseases. Phytother. Res. 32 (11), 2202–2213. 10.1002/ptr.6171 30080294

[B2] Ahmad I. Irfan S. Ali B. M. Kamli H. Ali S. P. Begum N. (2021). The SMAC mimetic AT-101 exhibits anti-tumor and anti-metastasis activity in lung adenocarcinoma cells by the IAPs/caspase-dependent apoptosis and p65-NFƙB cross-talk. Iran. J. Basic Med. Sci. 24 (7), 969–977. 10.22038/ijbms.2021.56400.12586 34712428 PMC8528260

[B3] Aiello P. Sharghi M. Mansourkhani S. M. Ardekan A. P. Jouybari L. Daraei N. (2019). Medicinal plants in the prevention and treatment of colon cancer. Oxid. Med. Cell Longev. 2019, 2075614. 10.1155/2019/2075614 32377288 PMC7187726

[B4] Ailawadhi S. Parrondo R. D. Dutta N. Han B. Ciccio G. Cherukuri Y. (2023). AT-101 enhances the antitumor activity of lenalidomide in patients with multiple myeloma. Cancers (Basel) 15 (2), 477. 10.3390/cancers15020477 36672426 PMC9857228

[B5] Aktepe N. Yukselten Y. (2022). Induction of apoptosis in human hormone-refractory prostate cancer cell lines by using resveratrol in combination with AT-101. Andrologia 54 (1), e14267. 10.1111/and.14267 34633104

[B6] Ammendolia I. Mannucci C. Cardia L. Calapai G. Gangemi S. Esposito E. (2023). Pharmacovigilance on cannabidiol as an antiepileptic agent. Front. Pharmacol. 14, 1091978. 10.3389/fphar.2023.1091978 36843933 PMC9950105

[B7] Arvanitis C. D. Ferraro G. B. Jain R. K. (2020). The blood-brain barrier and blood-tumour barrier in brain tumours and metastases. Nat. Rev. Cancer 20 (1), 26–41. 10.1038/s41568-019-0205-x 31601988 PMC8246629

[B8] Bachari A. Piva T. J. Salami S. A. Jamshidi N. Mantri N. (2020). Roles of cannabinoids in melanoma: Evidence from *in vivo* studies. Int. J. Mol. Sci. 21 (17), 6040. 10.3390/ijms21176040 32839414 PMC7503316

[B9] Bai S. Wen W. Hou X. Wu J. Yi L. Zhi Y. (2021). Inhibitory effect of sinomenine on lung cancer cells via negative regulation of α7 nicotinic acetylcholine receptor. J. Leukoc. Biol. 109 (4), 843–852. 10.1002/JLB.6MA0720-344RRR 32726882

[B10] Bai Y. Chen J. Hu W. Wang L. Wu Y. Yu S. (2022). Silibinin therapy improves cholangiocarcinoma outcomes by regulating ERK/mitochondrial pathway. Front. Pharmacol. 13, 847905. 10.3389/fphar.2022.847905 35401195 PMC8983842

[B11] Banelli B. Forlani A. Allemanni G. Morabito A. Pistillo M. P. Romani M. (2017). MicroRNA in glioblastoma: An overview. Int. J. Genomics 2017, 7639084. 10.1155/2017/7639084 29234674 PMC5695025

[B12] Batara D. Choi M. C. Shin H. U. Kim H. Kim S. H. (2021). Friend or foe: Paradoxical roles of autophagy in gliomagenesis. Cells 10 (6), 1411. 10.3390/cells10061411 34204169 PMC8227518

[B13] Batoon L. Koh A. J. Kannan R. Mccauley L. K. Roca H. (2023). Caspase-9 driven murine model of selective cell apoptosis and efferocytosis. Cell Death Dis. 14 (1), 58. 10.1038/s41419-023-05594-6 36693838 PMC9873735

[B14] Baur J. A. Sinclair D. A. (2006). Therapeutic potential of resveratrol: The *in vivo* evidence. Nat. Rev. Drug Discov. 5 (6), 493–506. 10.1038/nrd2060 16732220

[B15] Benlloch M. Obrador E. Valles S. L. Rodriguez M. L. Sirerol J. A. Alcacer J. (2016). Pterostilbene decreases the antioxidant defenses of aggressive cancer cells *in vivo*: A physiological glucocorticoids- and nrf2-dependent mechanism. Antioxid. Redox Signal 24 (17), 974–990. 10.1089/ars.2015.6437 26651028 PMC4921902

[B16] Benvenuto M. Mattera R. Sticca J. I. Rossi P. Cipriani C. Giganti M. G. (2018). Effect of the BH3 mimetic polyphenol (-)-Gossypol (AT-101) on the *in vitro* and *in vivo* growth of malignant mesothelioma. Front. Pharmacol. 9, 1269. 10.3389/fphar.2018.01269 30459622 PMC6232343

[B17] Bernardini J. P. Lazarou M. Dewson G. (2017). Parkin and mitophagy in cancer. Oncogene 36 (10), 1315–1327. 10.1038/onc.2016.302 27593930

[B18] Bhargava P. Schnellmann R. G. (2017). Mitochondrial energetics in the kidney. Nat. Rev. Nephrol. 13 (10), 629–646. 10.1038/nrneph.2017.107 28804120 PMC5965678

[B19] Biswas S. Dodwadkar N. S. Deshpande P. P. Torchilin V. P. (2012). Liposomes loaded with paclitaxel and modified with novel triphenylphosphonium-PEG-PE conjugate possess low toxicity, target mitochondria and demonstrate enhanced antitumor effects *in vitro* and *in vivo* . J. Control Release 159 (3), 393–402. 10.1016/j.jconrel.2012.01.009 22286008 PMC3348446

[B20] Boatright K. M. Salvesen G. S. (2003). Mechanisms of caspase activation. Curr. Opin. Cell Biol. 15 (6), 725–731. 10.1016/j.ceb.2003.10.009 14644197

[B21] Boulos J. C. Rahama M. Hegazy M. F. Efferth T. (2019). Shikonin derivatives for cancer prevention and therapy. Cancer Lett. 459, 248–267. 10.1016/j.canlet.2019.04.033 31132429

[B22] Bracht J. Karachaliou N. Berenguer J. Pedraz-Valdunciel C. Filipska M. Codony-Servat C. (2019). Osimertinib and pterostilbene in EGFR-mutation-positive non-small cell lung cancer (NSCLC). Int. J. Biol. Sci. 15 (12), 2607–2614. 10.7150/ijbs.32889 31754333 PMC6854375

[B23] Bravo-San P. J. Kroemer G. Galluzzi L. (2017). Autophagy and mitophagy in cardiovascular disease. Circ. Res. 120 (11), 1812–1824. 10.1161/circresaha.117.311082 28546358

[B24] Bulut G. Atmaca H. Karaca B. (2020). Trastuzumab in combination with AT-101 induces cytotoxicity and apoptosis in Her2 positive breast cancer cells. Future Oncol. 16 (3), 4485–4495. 10.2217/fon-2019-0521 31829029

[B25] Cammarata F. P. Torrisi F. Vicario N. Bravata V. Stefano A. Salvatorelli L. (2023). Proton boron capture therapy (PBCT) induces cell death and mitophagy in a heterotopic glioblastoma model. Commun. Biol. 6 (1), 388. 10.1038/s42003-023-04770-w 37031346 PMC10082834

[B26] Cao W. Chen G. Wu L. Yu K. N. Sun M. Yang M. (2023). Ionizing radiation triggers the antitumor immunity by inducing gasdermin E-mediated pyroptosis in tumor cells. Int. J. Radiat. Oncol. Biol. Phys. 115 (2), 440–452. 10.1016/j.ijrobp.2022.07.1841 35918054

[B27] Catalgol B. Batirel S. Taga Y. Ozer N. K. (2012). Resveratrol: French paradox revisited. Front. Pharmacol. 3141, 141. 10.3389/fphar.2012.00141 PMC3398412 22822401

[B28] Chang J. Rimando A. Pallas M. Camins A. Porquet D. Reeves J. (2012). Low-dose pterostilbene, but not resveratrol, is a potent neuromodulator in aging and Alzheimer's disease. Neurobiol. Aging 33 (9), 2062–2071. 10.1016/j.neurobiolaging.2011.08.015 21982274

[B29] Chen M. Yu S. (2019). Lipophilic grape seed proanthocyanidin exerts anti-proliferative and pro-apoptotic effects on PC3 human prostate cancer cells and suppresses PC3 xenograft tumor growth *in vivo* . J. Agric. Food Chem. 67 (1), 229–235. 10.1021/acs.jafc.8b05936 30562012

[B30] Chen P. H. Chang C. K. Shih C. M. Cheng C. H. Lin C. W. Lee C. C. (2016). The miR-204-3p-targeted IGFBP2 pathway is involved in xanthohumol-induced glioma cell apoptotic death. Neuropharmacology 110, 362–375. 10.1016/j.neuropharm.2016.07.038 27487563

[B31] Chen X. Lu C. Duan Y. Huang Y. (2022). Recent advancements in drug delivery of sinomenine, A disease-modifying anti-rheumatic drug. Pharmaceutics 14, 2820. 10.3390/pharmaceutics14122820 36559313 PMC9781253

[B32] Chen X. Zhang M. Gan H. Wang H. Lee J. H. Fang D. (2018). A novel enhancer regulates MGMT expression and promotes temozolomide resistance in glioblastoma. Nat. Commun. 9 (1), 2949. 10.1038/s41467-018-05373-4 30054476 PMC6063898

[B33] Chen Y. T. Huang Z. Y. Tang H. H. Kuo W. T. Wu S. Y. Lan S. H. (2020). Pterostilbene sensitizes cisplatin-resistant human bladder cancer cells with oncogenic HRAS. Cancers (Basel) 12 (10), 2869. 10.3390/cancers12102869 33036162 PMC7650649

[B34] Cheng Y. M. Tsai C. C. Hsu Y. C. (2016). Sulforaphane, a dietary isothiocyanate, induces G₂/M arrest in cervical cancer cells through CyclinB1 downregulation and gadd45β/CDC2 association. Int. J. Mol. Sci. 17 (9), 1530. 10.3390/ijms17091530 27626412 PMC5037805

[B35] Constantin M. Choi A. J. Cloonan S. M. Ryter S. W. (2012). Therapeutic potential of heme oxygenase-1/carbon monoxide in lung disease. Int. J. Hypertens. 2012, 859235. 10.1155/2012/859235 22518295 PMC3296197

[B36] Cook K. L. Warri A. Soto-Pantoja D. R. Clarke P. A. Cruz M. I. Zwart A. (2014). Hydroxychloroquine inhibits autophagy to potentiate antiestrogen responsiveness in ER+ breast cancer. Clin. Cancer Res. 20 (12), 3222–3232. 10.1158/1078-0432.CCR-13-3227 24928945 PMC4073207

[B37] Desagher S. Martinou J. C. (2000). Mitochondria as the central control point of apoptosis. Trends Cell Biol. 10 (9), 369–377. 10.1016/s0962-8924(00)01803-1 10932094

[B38] Di Meo S. Venditti P. Napolitano G. (2022). Physiological and pathological role of ROS: Benefits and limitations of antioxidant treatment 2.0. Int. J. Mol. Sci. 23 (16), 9437. 10.3390/ijms23169437 36012701 PMC9409188

[B39] Di Rita A. D'Acunzo P. Simula L. Campello S. Strappazzon F. Cecconi F. (2018). AMBRA1-Mediated mitophagy counteracts oxidative stress and apoptosis induced by neurotoxicity in human neuroblastoma SH-SY5Y cells. Front. Cell Neurosci. 12, 92. 10.3389/fncel.2018.00092 29755319 PMC5932353

[B40] Feng J. Wang C. Liu T. Li J. Wu L. Yu Q. (2019). Procyanidin B2 inhibits the activation of hepatic stellate cells and angiogenesis via the Hedgehog pathway during liver fibrosis. J. Cell Mol. Med. 23 (9), 6479–6493. 10.1111/jcmm.14543 31328391 PMC6714206

[B41] Feng Y. Yang Y. Fan C. Di S. Hu W. Jiang S. (2016). Pterostilbene inhibits the growth of human esophageal cancer cells by regulating endoplasmic reticulum stress. Cell Physiol. Biochem. 38 (3), 1226–1244. 10.1159/000443071 26982591

[B42] Festa M. Capasso A. D'Acunto C. W. Masullo M. Rossi A. G. Pizza C. (2011). Xanthohumol induces apoptosis in human malignant glioblastoma cells by increasing reactive oxygen species and activating MAPK pathways. J. Nat. Prod. 74 (12), 2505–2513. 10.1021/np200390x 22111577

[B43] Finucane D. M. Bossy-Wetzel E. Waterhouse N. J. Cotter T. G. Green D. R. (1999). Bax-induced caspase activation and apoptosis via cytochrome c release from mitochondria is inhibitable by Bcl-xL. J. Biol. Chem. 274 (4), 2225–2233. 10.1074/jbc.274.4.2225 9890985

[B44] Fu G. Yin G. Niu T. Wu W. Han H. Chen H. (2021). A novel ratiometric fluorescent probe for the detection of mitochondrial pH dynamics during cell damage. Analyst 146 (2), 620–627. 10.1039/d0an01240h 33188671

[B45] Gao H. Liu Z. Xu W. Wang Q. Zhang C. Ding Y. (2021). Pterostilbene promotes mitochondrial apoptosis and inhibits proliferation in glioma cells. Sci. Rep. 11 (1), 6381. 10.1038/s41598-021-85908-w 33737656 PMC7973728

[B46] Gao X. Sun B. Hou Y. Liu L. Sun J. Xu F. (2022). Anti-breast cancer sinomenine derivatives via mechanisms of apoptosis induction and metastasis reduction. J. Enzyme Inhib. Med. Chem. 37 (1), 1870–1883. 10.1080/14756366.2022.2096020 35801430 PMC9272937

[B47] Genovese T. Cordaro M. Siracusa R. Impellizzeri D. Caudullo S. Raffone E. (2022). Molecular and biochemical mechanism of cannabidiol in the management of the inflammatory and oxidative processes associated with endometriosis. Int. J. Mol. Sci. 23 (10), 5427. 10.3390/ijms23105427 35628240 PMC9141153

[B48] Gorbunova A. S. Yapryntseva M. A. Denisenko T. V. Zhivotovsky B. (2020). BNIP3 in lung cancer: To kill or rescue? Cancers (Basel) 12 (11), 3390. 10.3390/cancers12113390 33207677 PMC7697772

[B49] Gorick C. M. Sheybani N. D. Curley C. T. Price R. J. (2018). Listening in on the microbubble crowd: Advanced acoustic monitoring for improved control of blood-brain barrier opening with focused ultrasound. Theranostics 8 (11), 2988–2991. 10.7150/thno.26025 29897053 PMC5996352

[B50] Grace M. S. Bonvini S. J. Belvisi M. G. Mcintyre P. (2017). Modulation of the TRPV4 ion channel as a therapeutic target for disease. Pharmacol. Ther. 177, 9–22. 10.1016/j.pharmthera.2017.02.019 28202366

[B51] Gu J. Rauniyar S. Wang Y. Zhan W. Ye C. Ji S. (2021). Chrysophanol induced glioma cells apoptosis via activation of mitochondrial apoptosis pathway. Bioengineered 12 (1), 6855–6868. 10.1080/21655979.2021.1972079 34519612 PMC8806913

[B52] Guo C. He J. Song X. Tan L. Wang M. Jiang P. (2019). Pharmacological properties and derivatives of shikonin-A review in recent years. Pharmacol. Res. 149, 104463. 10.1016/j.phrs.2019.104463 31553936

[B53] Guo Q. L. Dai X. L. Yin M. Y. Cheng H. W. Qian H. S. Wang H. (2022). Nanosensitizers for sonodynamic therapy for glioblastoma multiforme: Current progress and future perspectives. Mil. Med. Res. 9 (1), 26. 10.1186/s40779-022-00386-z 35676737 PMC9178901

[B54] Habib H. M. El-Fakharany E. M. Kheadr E. Ibrahim W. H. (2022). Grape seed proanthocyanidin extract inhibits DNA and protein damage and labile iron, enzyme, and cancer cell activities. Sci. Rep. 12 (1), 12393. 10.1038/s41598-022-16608-2 35859159 PMC9300616

[B55] Harandi-Zadeh S. Boycott C. Beetch M. Yang T. Martin B. Ren K. (2021). Pterostilbene changes epigenetic marks at enhancer regions of oncogenes in breast cancer cells. Antioxidants (Basel) 10 (8), 1232. 10.3390/antiox10081232 34439480 PMC8388921

[B56] He X. Maimaiti M. Jiao Y. Meng X. Li H. (2018). Sinomenine induces G1-phase cell cycle arrest and apoptosis in malignant glioma cells via downregulation of sirtuin 1 and induction of p53 acetylation. Technol. Cancer Res. Treat. 17, 1533034618770305. 10.1177/1533034618770305 29756546 PMC5952277

[B57] Hegi M. E. Genbrugge E. Gorlia T. Stupp R. Gilbert M. R. Chinot O. L. (2019). MGMT promoter methylation cutoff with safety margin for selecting glioblastoma patients into trials omitting temozolomide: A pooled analysis of four clinical trials. Clin. Cancer Res. 25 (6), 1809–1816. 10.1158/1078-0432.CCR-18-3181 30514777 PMC8127866

[B58] Hemani R. Patel I. Inamdar N. Campanelli G. Donovan V. Kumar A. (2022). Dietary pterostilbene for MTA1-targeted interception in high-risk premalignant prostate cancer. Cancer Prev. Res. (Phila) 15 (2), 87–100. 10.1158/1940-6207.CAPR-21-0242 34675064 PMC8828670

[B59] Ho K. H. Chang C. K. Chen P. H. Wang Y. J. Chang W. C. Chen K. C. (2018). miR-4725-3p targeting stromal interacting molecule 1 signaling is involved in xanthohumol inhibition of glioma cell invasion. J. Neurochem. 146 (3), 269–288. 10.1111/jnc.14459 29747239

[B60] Ho K. H. Kuo T. C. Lee Y. T. Chen P. H. Shih C. M. Cheng C. H. (2020). Xanthohumol regulates miR-4749-5p-inhibited RFC2 signaling in enhancing temozolomide cytotoxicity to glioblastoma. Life Sci. 254, 117807. 10.1016/j.lfs.2020.117807 32422304

[B61] Ho K. H. Shih C. M. Liu A. J. Chen K. C. (2022). Hypoxia-inducible lncRNA MIR210HG interacting with OCT1 is involved in glioblastoma multiforme malignancy. Cancer Sci. 113 (2), 540–552. 10.1111/cas.15240 34897892 PMC8819343

[B62] Hosseinabadi T. Lorigooini Z. Tabarzad M. Salehi B. Rodrigues C. F. Martins N. (2019). Silymarin antiproliferative and apoptotic effects: Insights into its clinical impact in various types of cancer. Phytother. Res. 33 (11), 2849–2861. 10.1002/ptr.6470 31407422

[B63] Hou S. Song Y. Sun D. Zhu S. Wang Z. (2021). Xanthohumol-induced rat glioma C6 cells death by triggering mitochondrial stress. Int. J. Mol. Sci. 22 (9), 4506. 10.3390/ijms22094506 33925918 PMC8123451

[B64] Hu C. Niestroj M. Yuan D. Chang S. Chen J. (2015). Treating cancer stem cells and cancer metastasis using glucose-coated gold nanoparticles. Int. J. Nanomedicine 10, 2065–2077. 10.2147/IJN.S72144 25844037 PMC4368028

[B65] Huang H. Zhang S. Li Y. Liu Z. Mi L. Cai Y. (2021a). Suppression of mitochondrial ROS by prohibitin drives glioblastoma progression and therapeutic resistance. Nat. Commun. 12 (1), 3720. 10.1038/s41467-021-24108-6 34140524 PMC8211793

[B66] Huang M. Zhang D. Wu J. Y. Xing K. Yeo E. Li C. (2020). Wnt-mediated endothelial transformation into mesenchymal stem cell-like cells induces chemoresistance in glioblastoma. Sci. Transl. Med. 12, eaay7522. 10.1126/scitranslmed.aay7522 32102932 PMC7261487

[B67] Huang R. Zhou P. K. (2021). DNA damage repair: Historical perspectives, mechanistic pathways and clinical translation for targeted cancer therapy. Signal Transduct. Target Ther. 6 (1), 254. 10.1038/s41392-021-00648-7 34238917 PMC8266832

[B68] Huang T. Xu T. Wang Y. Zhou Y. Yu D. Wang Z. (2021b). Cannabidiol inhibits human glioma by induction of lethal mitophagy through activating TRPV4. Autophagy 17 (11), 3592–3606. 10.1080/15548627.2021.1885203 33629929 PMC8632311

[B69] Huangfu M. Wei R. Wang J. Qin J. Yu D. Guan X. (2021). Osthole induces necroptosis via ROS overproduction in glioma cells. FEBS Open Bio 11 (2), 456–467. 10.1002/2211-5463.13069 PMC7876487 33350608

[B70] Hull T. D. Boddu R. Guo L. Tisher C. C. Traylor A. M. Patel B. (2016). Heme oxygenase-1 regulates mitochondrial quality control in the heart. JCI Insight 1 (2), e85817. 10.1172/jci.insight.85817 27110594 PMC4838906

[B71] Jahanafrooz Z. Motamed N. Rinner B. Mokhtarzadeh A. Baradaran B. (2018). Silibinin to improve cancer therapeutic, as an apoptotic inducer, autophagy modulator, cell cycle inhibitor, and microRNAs regulator. Life Sci. 213, 236–247. 10.1016/j.lfs.2018.10.009 30308184

[B72] Jeong S. Yun H. K. Jeong Y. A. Jo M. J. Kang S. H. Kim J. L. (2019). Cannabidiol-induced apoptosis is mediated by activation of Noxa in human colorectal cancer cells. Cancer Lett. 447, 12–23. 10.1016/j.canlet.2019.01.011 30660647

[B73] Jhaveri A. Deshpande P. Pattni B. Torchilin V. (2018). Transferrin-targeted, resveratrol-loaded liposomes for the treatment of glioblastoma. J. Control Release 277, 89–101. 10.1016/j.jconrel.2018.03.006 29522834 PMC5911193

[B74] Karimi-Haghighi S. Razavi Y. Iezzi D. Scheyer A. F. Manzoni O. Haghparast A. (2022). Cannabidiol and substance use disorder: Dream or reality. Neuropharmacology 207, 108948. 10.1016/j.neuropharm.2022.108948 35032495 PMC9157244

[B75] Katayama H. Hama H. Nagasawa K. Kurokawa H. Sugiyama M. Ando R. (2020). Visualizing and modulating mitophagy for therapeutic studies of neurodegeneration. Cell 181 (5), 1176–1187.e16. 10.1016/j.cell.2020.04.025 32437660

[B76] Kerr J. S. Adriaanse B. A. Greig N. H. Mattson M. P. Cader M. Z. Bohr V. A. (2017). Mitophagy and Alzheimer's disease: Cellular and molecular mechanisms. Trends Neurosci. 40 (3), 151–166. 10.1016/j.tins.2017.01.002 28190529 PMC5341618

[B77] Khodadadi H. Salles E. L. Alptekin A. Mehrabian D. Rutkowski M. Arbab A. S. (2021). Inhalant cannabidiol inhibits glioblastoma progression through regulation of tumor microenvironment. Cannabis Cannabinoid Res. 10.1089/can.2021.0098 PMC10589502 34918964

[B78] Kim H. Y. Lee B. I. Jeon J. H. Kim D. K. Kang S. G. Shim J. K. (2019). Gossypol suppresses growth of temozolomide-resistant glioblastoma tumor spheres. Biomolecules 9 (10), 595. 10.3390/biom9100595 31658771 PMC6843396

[B79] Kiskova T. Mungenast F. Suvakova M. Jager W. Thalhammer T. (2019). Future aspects for cannabinoids in breast cancer therapy. Int. J. Mol. Sci. 20 (7), 1673. 10.3390/ijms20071673 30987191 PMC6479799

[B80] Kleih M. Bopple K. Dong M. Gaissler A. Heine S. Olayioye M. A. (2019). Direct impact of cisplatin on mitochondria induces ROS production that dictates cell fate of ovarian cancer cells. Cell Death Dis. 10 (11), 851. 10.1038/s41419-019-2081-4 31699970 PMC6838053

[B81] Kumar V. Haldar S. Das N. S. Ghosh S. Dhankhar P. Sircar D. (2021). Pterostilbene-isothiocyanate inhibits breast cancer metastasis by selectively blocking IKK-β/NEMO interaction in cancer cells. Biochem. Pharmacol. 192, 114717. 10.1016/j.bcp.2021.114717 34352281

[B82] Kuo C. Y. Chiu V. Hsieh P. C. Huang C. Y. Huang S. J. Tzeng I. S. (2020). Chrysophanol attenuates Hepatitis B virus X protein-induced hepatic stellate cell fibrosis by regulating endoplasmic reticulum stress and ferroptosis. J. Pharmacol. Sci. 144 (3), 172–182. 10.1016/j.jphs.2020.07.014 32811746

[B83] Lah T. T. Novak M. Breznik B. (2020). Brain malignancies: Glioblastoma and brain metastases. Semin. Cancer Biol. 60, 262–273. 10.1016/j.semcancer.2019.10.010 31654711

[B84] Landhuis E. (2017). Ultrasound for the brain. Nature 551 (7679), 257–259. 10.1038/d41586-017-05479-7 29120442

[B85] Langen U. H. Ayloo S. Gu C. (2019). Development and cell biology of the blood-brain barrier. Annu. Rev. Cell Dev. Biol. 35, 591–613. 10.1146/annurev-cellbio-100617-062608 31299172 PMC8934576

[B86] Leao B. M. Pinheiro D. Borges B. (2021). Mitochondrial DNA alterations in glioblastoma (GBM). Int. J. Mol. Sci. 22 (11), 5855. 10.3390/ijms22115855 34072607 PMC8199454

[B87] Lee H. S. Tamia G. Song H. J. Amarakoon D. Wei C. I. Lee S. H. (2022a). Cannabidiol exerts anti-proliferative activity via a cannabinoid receptor 2-dependent mechanism in human colorectal cancer cells. Int. Immunopharmacol. 108, 108865. 10.1016/j.intimp.2022.108865 35598400

[B88] Lee J. Kim E. Chong K. Ryu S. W. Kim C. Choi K. (2022b). Atypical induction of HIF-1α expression by pericellular Notch1 signaling suffices for the malignancy of glioblastoma multiforme cells. Cell Mol. Life Sci. 79 (10), 537. 10.1007/s00018-022-04529-2 36183290 PMC9527190

[B89] Lee J. Y. Park J. Y. Kim D. H. Kim H. D. Ji Y. J. Seo K. H. (2020). Erigeron annuus protects PC12 neuronal cells from oxidative stress induced by ROS-mediated apoptosis. Evid. Based Complement. Altern. Med. 2020, 3945194. 10.1155/2020/3945194 PMC6970001 31998396

[B90] Levy J. Towers C. G. Thorburn A. (2017). Targeting autophagy in cancer. Nat. Rev. Cancer 17 (9), 528–542. 10.1038/nrc.2017.53 28751651 PMC5975367

[B91] Li C. Zhang L. Liu C. He X. Chen M. Chen J. (2022a). Roles of hydrogen gas in plants under abiotic stress: Current knowledge and perspectives. Antioxidants (Basel) 11 (2), 1999. 10.3390/antiox11101999 36290722 PMC9598357

[B92] Li S. Zhang J. Liu C. Wang Q. Yan J. Hui L. (2021). The role of mitophagy in regulating cell death. Oxid. Med. Cell Longev. 2021, 6617256. 10.1155/2021/6617256 34113420 PMC8154277

[B93] Li W. Hua B. Saud S. M. Lin H. Hou W. Matter M. S. (2015). Berberine regulates AMP-activated protein kinase signaling pathways and inhibits colon tumorigenesis in mice. Mol. Carcinog. 54 (10), 1096–1109. 10.1002/mc.22179 24838344 PMC4504840

[B94] Li X. Chen W. Huang L. Zhu M. Zhang H. Si Y. (2022b). Sinomenine hydrochloride suppresses the stemness of breast cancer stem cells by inhibiting Wnt signaling pathway through down-regulation of WNT10B. Pharmacol. Res. 179, 106222. 10.1016/j.phrs.2022.106222 35413424

[B95] Li X. He S. Ma B. (2020). Autophagy and autophagy-related proteins in cancer. Mol. Cancer 19 (1), 12. 10.1186/s12943-020-1138-4 31969156 PMC6975070

[B96] Li-Weber M. (2013). Targeting apoptosis pathways in cancer by Chinese medicine. Cancer Lett. 332 (2), 304–312. 10.1016/j.canlet.2010.07.015 20685036

[B97] Lin J. H. Yang K. T. Lee W. S. Ting P. C. Luo Y. P. Lin D. J. (2022a). Xanthohumol protects the rat myocardium against ischemia/reperfusion injury-induced ferroptosis. Oxid. Med. Cell Longev. 2022, 9523491. 10.1155/2022/9523491 35082973 PMC8786462

[B98] Lin K. Gueble S. E. Sundaram R. K. Huseman E. D. Bindra R. S. Herzon S. B. (2022b). Mechanism-based design of agents that selectively target drug-resistant glioma. Science 377 (6605), 502–511. 10.1126/science.abn7570 35901163 PMC9502022

[B99] Lin Y. Yi O. Hu M. Hu S. Su Z. Liao J. (2022c). Multifunctional nanoparticles of sinomenine hydrochloride for treat-to-target therapy of rheumatoid arthritis via modulation of proinflammatory cytokines. J. Control Release 348, 42–56. 10.1016/j.jconrel.2022.05.016 35569587

[B100] Lindenboim L. Yuan J. Stein R. (2000). Bcl-xS and Bax induce different apoptotic pathways in PC12 cells. Oncogene 19 (14), 1783–1793. 10.1038/sj.onc.1203495 10777212

[B101] Linder B. Wehle A. Hehlgans S. Bonn F. Dikic I. Rodel F. (2019). Arsenic trioxide and (-)-Gossypol synergistically target glioma stem-like cells via inhibition of hedgehog and notch signaling. Cancers (Basel) 11 (3), 350. 10.3390/cancers11030350 30871073 PMC6468469

[B102] Liu B. Cao Y. Wang D. Zhou Y. Zhang P. Wu J. (2021). Zhen-Wu-Tang induced mitophagy to protect mitochondrial function in chronic glomerulonephritis via PI3K/AKT/mTOR and AMPK pathways. Front. Pharmacol. 12, 777670. 10.3389/fphar.2021.777670 35757387 PMC9231558

[B103] Liu B. Zhou J. Wang C. Chi Y. Wei Q. Fu Z. (2020a). LncRNA SOX2OT promotes temozolomide resistance by elevating SOX2 expression via ALKBH5-mediated epigenetic regulation in glioblastoma. Cell Death Dis. 11 (5), 384. 10.1038/s41419-020-2540-y 32439916 PMC7242335

[B104] Liu H. Zhang R. Zhang D. Zhang C. Zhang Z. Fu X. (2022). Cyclic RGD-decorated liposomal gossypol AT-101 targeting for enhanced antitumor effect. Int. J. Nanomedicine 17, 227–244. 10.2147/IJN.S341824 35068931 PMC8766252

[B105] Liu K. Zheng M. Lu R. Du J. Zhao Q. Li Z. (2020b). The role of CDC25C in cell cycle regulation and clinical cancer therapy: A systematic review. Cancer Cell Int. 20, 213. 10.1186/s12935-020-01304-w 32518522 PMC7268735

[B106] Liu Y. Liu C. Tan T. Li S. Tang S. Chen X. (2019). Sinomenine sensitizes human gastric cancer cells to cisplatin through negative regulation of PI3K/AKT/Wnt signaling pathway. Anticancer Drugs 30 (10), 983–990. 10.1097/CAD.0000000000000834 31609766 PMC6824511

[B107] Lopez-Valero I. Saiz-Ladera C. Torres S. Hernandez-Tiedra S. Garcia-Taboada E. Rodriguez-Fornes F. (2018). Targeting Glioma Initiating Cells with A combined therapy of cannabinoids and temozolomide. Biochem. Pharmacol. 157, 266–274. 10.1016/j.bcp.2018.09.007 30195736

[B108] Louis D. N. Perry A. Wesseling P. Brat D. J. Cree I. A. Figarella-Branger D. (2021). The 2021 WHO classification of tumors of the central nervous system: A summary. Neuro Oncol. 23 (8), 1231–1251. 10.1093/neuonc/noab106 34185076 PMC8328013

[B109] Lu B. Gong X. Wang Z. Q. Ding Y. Wang C. Luo T. F. (2017). Shikonin induces glioma cell necroptosis *in vitro* by ROS overproduction and promoting RIP1/RIP3 necrosome formation. Acta Pharmacol. Sin. 38 (11), 1543–1553. 10.1038/aps.2017.112 28816233 PMC5672068

[B110] Lu R. O. Ho W. S. (2020). Mitochondrial dysfunction, macrophage, and microglia in brain cancer. Front. Cell Dev. Biol. 8, 620788. 10.3389/fcell.2020.620788 33520994 PMC7843701

[B111] Ma X. Yu M. Hao C. Yang W. (2020). Shikonin induces tumor apoptosis in glioma cells via endoplasmic reticulum stress, and Bax/Bak mediated mitochondrial outer membrane permeability. J. Ethnopharmacol. 263, 113059. 10.1016/j.jep.2020.113059 32663591

[B112] Ma Z. Zhang X. Xu L. Liu D. Di S. Li W. (2019). Pterostilbene: Mechanisms of its action as oncostatic agent in cell models and *in vivo* studies. Pharmacol. Res. 145, 104265. 10.1016/j.phrs.2019.104265 31078746

[B113] Mahmoud A. M. Kostrzewa M. Marolda V. Cerasuolo M. Maccarinelli F. Coltrini D. (2023). Cannabidiol alters mitochondrial bioenergetics via VDAC1 and triggers cell death in hormone-refractory prostate cancer. Pharmacol. Res. 189, 106683. 10.1016/j.phrs.2023.106683 36736415

[B114] Maiti A. Hait N. C. (2021). Autophagy-mediated tumor cell survival and progression of breast cancer metastasis to the brain. J. Cancer 12 (4), 954–964. 10.7150/jca.50137 33442395 PMC7797661

[B115] Maiti P. Scott J. Sengupta D. Al-Gharaibeh A. Dunbar G. L. (2019). Curcumin and solid lipid curcumin particles induce autophagy, but inhibit mitophagy and the PI3K-Akt/mTOR pathway in cultured glioblastoma cells. Int. J. Mol. Sci. 20 (2), 399. 10.3390/ijms20020399 30669284 PMC6359162

[B116] Mao J. T. Xue B. Lu Q. Y. Lundmark L. Burns W. Yang J. (2023). Combinations of grape seed procyanidin extract and milk thistle silymarin extract against lung cancer - the role of MiR-663a and FHIT. Life Sci. 318, 121492. 10.1016/j.lfs.2023.121492 36775115

[B117] Mehner M. Kubelt C. Adamski V. Schmitt C. Synowitz M. Held-Feindt J. (2020). Combined treatment of AT101 and demethoxycurcumin yields an enhanced anti-proliferative effect in human primary glioblastoma cells. J. Cancer Res. Clin. Oncol. 146 (1), 117–126. 10.1007/s00432-019-03107-7 31844979 PMC11804512

[B118] Meng Y. Qiu L. Zeng X. Hu X. Zhang Y. Wan X. (2022). Targeting CRL4 suppresses chemoresistant ovarian cancer growth by inducing mitophagy. Signal Transduct. Target Ther. 7 (1), 388. 10.1038/s41392-022-01253-y 36481655 PMC9731993

[B119] Mess G. Anderson T. Kapoor S. Thombre R. Liang R. Derin E. (2023). Sonodynamic therapy for the treatment of glioblastoma multiforme in a mouse model using a portable benchtop focused ultrasound system. J. Vis. Exp. No. 192. 10.3791/65114 36847383

[B120] Meyer N. Zielke S. Michaelis J. B. Linder B. Warnsmann V. Rakel S. (2018). AT 101 induces early mitochondrial dysfunction and HMOX1 (heme oxygenase 1) to trigger mitophagic cell death in glioma cells. Autophagy 14 (10), 1693–1709. 10.1080/15548627.2018.1476812 29938581 PMC6135628

[B121] Milian L. Monleon-Guinot I. Sancho-Tello M. Galbis J. M. Cremades A. Almenar-Ordaz M. (2022). *In vitro* effect of d9-tetrahydrocannabinol and cannabidiol on cancer-associated fibroblasts isolated from lung cancer. Int. J. Mol. Sci. 23 (12), 6766. 10.3390/ijms23126766 35743206 PMC9223514

[B122] Misri S. Kaul K. Mishra S. Charan M. Verma A. K. Barr M. P. (2022). Cannabidiol inhibits tumorigenesis in cisplatin-resistant non-small cell lung cancer via TRPV2. Cancers (Basel) 14 (5), 1181. 10.3390/cancers14051181 35267489 PMC8909073

[B123] Molinaro A. M. Wiencke J. K. Warrier G. Koestler D. C. Chunduru P. Lee J. Y. (2022). Interactions of age and blood immune factors and noninvasive prediction of glioma survival. J. Natl. Cancer Inst. 114 (3), 446–457. 10.1093/jnci/djab195 34597382 PMC8902347

[B124] Muller C. Reggio P. H. (2020). An analysis of the putative CBD binding site in the ionotropic cannabinoid receptors. Front. Cell Neurosci. 14, 615811. 10.3389/fncel.2020.615811 33362478 PMC7755602

[B125] Neumann H. F. Frank J. Venturelli S. Egert S. (2022). Bioavailability and cardiometabolic effects of xanthohumol: Evidence from animal and human studies. Mol. Nutr. Food Res. 66 (6), e2100831. 10.1002/mnfr.202100831 34967501

[B126] Neves A. R. Lucio M. Lima J. L. Reis S. (2012). Resveratrol in medicinal chemistry: A critical review of its pharmacokinetics, drug-delivery, and membrane interactions. Curr. Med. Chem. 19 (11), 1663–1681. 10.2174/092986712799945085 22257059

[B127] Ni C. H. Chen P. Y. Lu H. F. Yang J. S. Huang H. Y. Wu S. H. (2012). Chrysophanol-induced necrotic-like cell death through an impaired mitochondrial ATP synthesis in Hep3B human liver cancer cells. Arch. Pharm. Res. 35 (5), 887–895. 10.1007/s12272-012-0514-z 22644856

[B128] Oberoi R. K. Parrish K. E. Sio T. T. Mittapalli R. K. Elmquist W. F. Sarkaria J. N. (2016). Strategies to improve delivery of anticancer drugs across the blood-brain barrier to treat glioblastoma. Neuro Oncol. 18 (1), 27–36. 10.1093/neuonc/nov164 26359209 PMC4677418

[B129] Ohgaki H. Kleihues P. (2005). Population-based studies on incidence, survival rates, and genetic alterations in astrocytic and oligodendroglial gliomas. J. Neuropathol. Exp. Neurol. 64 (6), 479–489. 10.1093/jnen/64.6.479 15977639

[B130] Ostrom Q. T. Cioffi G. Gittleman H. Patil N. Waite K. Kruchko C. (2019), CBTRUS statistical report: Primary brain and other central nervous system tumors diagnosed in the United States in 2012-2016. Neuro Oncol. 21, v1-v100. 10.1093/neuonc/noz150 31675094 PMC6823730

[B131] Panigrahi D. P. Praharaj P. P. Bhol C. S. Mahapatra K. K. Patra S. Behera B. P. (2020). The emerging, multifaceted role of mitophagy in cancer and cancer therapeutics. Semin. Cancer Biol. 66, 45–58. 10.1016/j.semcancer.2019.07.015 31351198

[B132] Park D. B. Park B. S. Kang H. M. Kim J. H. Kim I. R. (2022). Chrysophanol-induced autophagy disrupts apoptosis via the PI3K/Akt/mTOR pathway in oral squamous cell carcinoma cells. Med. Kaunas. 59 (1), 42. 10.3390/medicina59010042 PMC9864245 36676666

[B133] Park M. Chae H. D. Yun J. Jung M. Kim Y. S. Kim S. H. (2000). Constitutive activation of cyclin B1-associated cdc2 kinase overrides p53-mediated G2-M arrest. Cancer Res. 60 (3), 542–545. 10676633

[B134] Qu F. Wang P. Zhang K. Shi Y. Li Y. Li C. (2020). Manipulation of Mitophagy by "All-in-One" nanosensitizer augments sonodynamic glioma therapy. Autophagy 16 (8), 1413–1435. 10.1080/15548627.2019.1687210 31674265 PMC7480814

[B135] Qu X. Yu B. Zhu M. Li X. Ma L. Liu C. (2021). Sinomenine inhibits the growth of ovarian cancer cells through the suppression of mitosis by down-regulating the expression and the activity of CDK1. Onco Targets Ther. 14, 823–834. 10.2147/OTT.S284261 33574676 PMC7873025

[B136] Que F. Dai L. Zhou D. Lin Q. Zeng X. Yu L. (2019). AT-101 induces G1/G0 phase arrest via the beta-catenin/cyclin D1 signaling pathway in human esophageal cancer cells. Oncol. Rep. 41 (2), 1415–1423. 10.3892/or.2018.6876 30483745

[B137] Rademaker G. Boumahd Y. Peiffer R. Anania S. Wissocq T. Liegeois M. (2022). Myoferlin targeting triggers mitophagy and primes ferroptosis in pancreatic cancer cells. Redox Biol. 53, 102324. 10.1016/j.redox.2022.102324 35533575 PMC9096673

[B138] Renner O. Mayer M. Leischner C. Burkard M. Berger A. Lauer U. M. (2022). Preclinical efficacy and toxicity analysis of the pan-histone deacetylase inhibitor gossypol for the therapy of colorectal cancer or hepatocellular carcinoma. Pharm. (Basel) 15 (2), 438. 10.3390/ph15040438 PMC9028974 35455435

[B139] Rimando A. M. Kalt W. Magee J. B. Dewey J. Ballington J. R. (2004). Resveratrol, pterostilbene, and piceatannol in vaccinium berries. J. Agric. Food Chem. 52 (15), 4713–4719. 10.1021/jf040095e 15264904

[B140] Ruiz M. J. Fernandez M. Pico Y. Manes J. Asensi M. Carda C. (2009). Dietary administration of high doses of pterostilbene and quercetin to mice is not toxic. J. Agric. Food Chem. 57 (8), 3180–3186. 10.1021/jf803579e 19292443

[B141] Sarkaria J. N. Hu L. S. Parney I. F. Pafundi D. H. Brinkmann D. H. Laack N. N. (2018). Is the blood-brain barrier really disrupted in all glioblastomas? A critical assessment of existing clinical data. Neuro Oncol. 20 (2), 184–191. 10.1093/neuonc/nox175 29016900 PMC5777482

[B142] Senft D. Ronai Z. A. (2016). Regulators of mitochondrial dynamics in cancer. Curr. Opin. Cell Biol. 39, 3943–3952. 10.1016/j.ceb.2016.02.001 PMC4828329 26896558

[B143] Shin H. J. Han J. M. Choi Y. S. Jung H. J. (2020). Pterostilbene suppresses both cancer cells and cancer stem-like cells in cervical cancer with superior bioavailability to resveratrol. Molecules 25 (1), 228. 10.3390/molecules25010228 31935877 PMC6982958

[B144] Si L. Fu J. Liu W. Hayashi T. Nie Y. Mizuno K. (2020). Silibinin inhibits migration and invasion of breast cancer MDA-MB-231 cells through induction of mitochondrial fusion. Mol. Cell Biochem. 463 (1-2), 189–201. 10.1007/s11010-019-03640-6 31612353

[B145] Song W. H. Zuidema D. Yi Y. J. Zigo M. Zhang Z. Sutovsky M. (2021). Mammalian cell-free system recapitulates the early events of post-fertilization sperm mitophagy. Cells 10 (9), 2450. 10.3390/cells10092450 34572103 PMC8466530

[B146] Steeg P. S. (2021). The blood-tumour barrier in cancer biology and therapy. Nat. Rev. Clin. Oncol. 18 (11), 696–714. 10.1038/s41571-021-00529-6 34253912

[B147] Su S. Wu J. Gao Y. Luo Y. Yang D. Wang P. (2020). The pharmacological properties of chrysophanol, the recent advances. Biomed. Pharmacother. 125, 110002. 10.1016/j.biopha.2020.110002 32066044

[B148] Su Y. C. Davuluri G. V. Chen C. H. Shiau D. C. Chen C. C. Chen C. L. (2016). Galectin-1-Induced autophagy facilitates cisplatin resistance of hepatocellular carcinoma. PLoS One 11 (2), e0148408. 10.1371/journal.pone.0148408 26859293 PMC4747500

[B149] Sun Y. Yu J. Liu X. Zhang C. Cao J. Li G. (2018a). Oncosis-like cell death is induced by berberine through ERK1/2-mediated impairment of mitochondrial aerobic respiration in gliomas. Biomed. Pharmacother. 102, 699–710. 10.1016/j.biopha.2018.03.132 29604589

[B150] Sun Z. Zheng L. Liu X. Xing W. Liu X. (2018b). Sinomenine inhibits the growth of melanoma by enhancement of autophagy via PI3K/AKT/mTOR inhibition. Drug Des. Devel Ther. 12, 2413–2421. 10.2147/DDDT.S155798 PMC6084074 30122899

[B151] Szklener K. Mazurek M. Wieteska M. Waclawska M. Bilski M. Mandziuk S. (2022). New directions in the therapy of glioblastoma. Cancers (Basel) 14 (21), 5377. 10.3390/cancers14215377 36358795 PMC9655599

[B152] Tan K. T. Chen P. W. Li S. Ke T. M. Lin S. H. Yang C. C. (2019). Pterostilbene inhibits lung squamous cell carcinoma growth *in vitro* and *in vivo* by inducing S phase arrest and apoptosis. Oncol. Lett. 18 (2), 1631–1640. 10.3892/ol.2019.10499 31423230 PMC6607121

[B153] Tang Q. Li W. Zheng X. Ren L. Liu J. Li S. (2020). MELK is an oncogenic kinase essential for metastasis, mitotic progression, and programmed death in lung carcinoma. Signal Transduct. Target Ther. 5 (1), 279. 10.1038/s41392-020-00288-3 33262323 PMC7708490

[B154] Tang W. Fan W. Lau J. Deng L. Shen Z. Chen X. (2019). Emerging blood-brain-barrier-crossing nanotechnology for brain cancer theranostics. Chem. Soc. Rev. 48 (11), 2967–3014. 10.1039/c8cs00805a 31089607

[B155] Taylor W. R. Stark G. R. (2001). Regulation of the G2/M transition by p53. Oncogene 20 (15), 1803–1815. 10.1038/sj.onc.1204252 11313928

[B156] Thannickal V. J. Fanburg B. L. (2000). Reactive oxygen species in cell signaling. Am. J. Physiol. Lung Cell Mol. Physiol. 279 (6), L1005–L1028. 10.1152/ajplung.2000.279.6.L1005 11076791

[B157] Tong C. Wang Y. Li J. Cen W. Zhang W. Zhu Z. (2021). Pterostilbene inhibits gallbladder cancer progression by suppressing the PI3K/Akt pathway. Sci. Rep. 11 (1), 4391. 10.1038/s41598-021-83924-4 33623100 PMC7902850

[B158] Tuli H. S. Mittal S. Aggarwal D. Parashar G. Parashar N. C. Upadhyay S. K. (2021). Path of silibinin from diet to medicine: A dietary polyphenolic flavonoid having potential anti-cancer therapeutic significance. Semin. Cancer Biol. 73, 196–218. 10.1016/j.semcancer.2020.09.014 33130037

[B159] Valenti C. Billi M. Pancrazi G. L. Calabria E. Armogida N. G. Tortora G. (2022). Biological effects of cannabidiol on human cancer cells: Systematic review of the literature. Pharmacol. Res. 181, 106267. 10.1016/j.phrs.2022.106267 35643249

[B160] Vara-Perez M. Felipe-Abrio B. Agostinis P. (2019). Mitophagy in cancer: A tale of adaptation. Cells 8 (5), 493. 10.3390/cells8050493 31121959 PMC6562743

[B161] Vara-Perez M. Rossi M. Van den Haute C. Maes H. Sassano M. L. Venkataramani V. (2021). BNIP3 promotes HIF-1α-driven melanoma growth by curbing intracellular iron homeostasis. EMBO J. 40 (10), e106214. 10.15252/embj.2020106214 33932034 PMC8126921

[B162] Volmar M. Cheng J. Alenezi H. Richter S. Haug A. Hassan Z. (2021). Cannabidiol converts NF-κB into a tumor suppressor in glioblastoma with defined antioxidative properties. Neuro Oncol. 23 (11), 1898–1910. 10.1093/neuonc/noab095 33864076 PMC8563328

[B163] Wang C. He C. Lu S. Wang X. Wang L. Liang S. (2020). Autophagy activated by silibinin contributes to glioma cell death via induction of oxidative stress-mediated BNIP3-dependent nuclear translocation of AIF. Cell Death Dis. 11 (8), 630. 10.1038/s41419-020-02866-3 32801360 PMC7429844

[B164] Wang H. Fang B. Peng B. Wang L. Xue Y. Bai H. (2021). Recent advances in chemical biology of mitochondria targeting. Front. Chem. 9, 683220. 10.3389/fchem.2021.683220 34012953 PMC8126688

[B165] Wang H. Zhu Z. Zhang G. Lin F. Liu Y. Zhang Y. (2019). AS1411 aptamer/hyaluronic acid-bifunctionalized microemulsion Co-loading shikonin and docetaxel for enhanced antiglioma therapy. J. Pharm. Sci. 108 (11), 3684–3694. 10.1016/j.xphs.2019.08.017 31465736

[B166] Wang J. Qiu X. Huang J. Zhuo Z. Chen H. Zeng R. (2022). Development and validation of a novel mitophagy-related gene prognostic signature for glioblastoma multiforme. BMC Cancer 22 (1), 644. 10.1186/s12885-022-09707-w 35692054 PMC9190154

[B167] Wang L. Zhan J. Huang W. (2020a). Grape seed proanthocyanidins induce apoptosis and cell cycle arrest of HepG2 cells accompanied by induction of the MAPK pathway and NAG-1. Antioxidants (Basel) 9 (12), 1200. 10.3390/antiox9121200 33260632 PMC7760884

[B168] Wang S. An J. Dong W. Wang X. Sheng J. Jia Y. (2020b). Glucose-coated berberine nanodrug for glioma therapy through mitochondrial pathway. Int. J. Nanomedicine 15, 7951–7965. 10.1038/s41419-020-02866-3 33116511 PMC7569050

[B169] Wang Y. Tang C. Cai J. Chen G. Zhang D. Zhang Z. (2018). PINK1/Parkin-mediated mitophagy is activated in cisplatin nephrotoxicity to protect against kidney injury. Cell Death Dis. 9 (11), 1113. 10.1038/s41419-018-1152-2 30385753 PMC6212494

[B170] Watson D. C. Bayik D. Storevik S. Moreino S. S. Sprowls S. A. Han J. (2023). GAP43-dependent mitochondria transfer from astrocytes enhances glioblastoma tumorigenicity. Nat. Cancer 4 (5), 648–664. 10.1038/s43018-023-00556-5 37169842 PMC10212766

[B171] Wawszczyk J. Jesse K. Smolik S. Kapral M. (2022). Mechanism of pterostilbene-induced cell death in HT-29 colon cancer cells. Molecules 27 (2), 369. 10.3390/molecules27020369 35056682 PMC8779997

[B172] Wong S. C. Kamarudin M. Naidu R. (2023). Anticancer mechanism of flavonoids on high-grade adult-type diffuse gliomas. Nutrients 15 (4), 797. 10.3390/nu15040797 36839156 PMC9964830

[B173] Xie H. Yin J. Shah M. H. Menefee M. E. Bible K. C. Reidy-Lagunes D. (2019a). A phase II study of the orally administered negative enantiomer of gossypol (AT-101), a BH3 mimetic, in patients with advanced adrenal cortical carcinoma. Invest. New Drugs 37 (4), 755–762. 10.1007/s10637-019-00797-1 31172443 PMC7515770

[B174] Xie L. Tang H. Song J. Long J. Zhang L. Li X. (2019b). Chrysophanol: A review of its pharmacology, toxicity and pharmacokinetics. J. Pharm. Pharmacol. 71 (10), 1475–1487. 10.1111/jphp.13143 31373015

[B175] Xu F. Li Q. Wang Z. Cao X. (2019). Sinomenine inhibits proliferation, migration, invasion and promotes apoptosis of prostate cancer cells by regulation of miR-23a. Biomed. Pharmacother. 112, 108592. 10.1016/j.biopha.2019.01.053 30784907

[B176] Xu H. Dong J. Hou J. Gao R. (2021a). Sinomenine inhibits the progression of bladder cancer cells by downregulating LncRNA-HEIH expression. Evid. Based Complement. Altern. Med. 2021, 4699529. 10.1155/2021/4699529 PMC8575624 34760016

[B177] Xu X. Liu Y. Wang L. He J. Zhang H. Chen X. (2009). Gambogic acid induces apoptosis by regulating the expression of Bax and Bcl-2 and enhancing caspase-3 activity in human malignant melanoma A375 cells. Int. J. Dermatol 48 (2), 186–192. 10.1111/j.1365-4632.2009.03946.x 19200201

[B178] Xu Y. Huang Y. Chen Y. Cao K. Liu Z. Wan Z. (2021b). Grape seed proanthocyanidins play the roles of radioprotection on normal lung and radiosensitization on lung cancer via differential regulation of the MAPK signaling pathway. J. Cancer 12 (10), 2844–2854. 10.7150/jca.49987 33854585 PMC8040900

[B179] Yang J. T. Li Z. L. Wu J. Y. Lu F. J. Chen C. H. (2014). An oxidative stress mechanism of shikonin in human glioma cells. PLoS One 9 (4), e94180. 10.1371/journal.pone.0094180 24714453 PMC3979747

[B180] Yang N. Gao J. Hou R. Xu X. Yang N. Huang S. (2021a). Grape seed proanthocyanidins inhibit migration and invasion of bladder cancer cells by reversing EMT through suppression of TGF-beta signaling pathway. Oxid. Med. Cell Longev. 2021, 5564312. 10.1155/2021/5564312 34354794 PMC8331280

[B181] Yang W. Feng Q. Li M. Su J. Wang P. Wang X. (2021b). Sinomenine suppresses development of hepatocellular carcinoma cells via inhibiting MARCH1 and AMPK/STAT3 signaling pathway. Front. Mol. Biosci. 8, 684262. 10.3389/fmolb.2021.684262 34179090 PMC8222788

[B182] Yang W. Wu P. F. Ma J. X. Liao M. J. Xu L. S. Yi L. (2020). TRPV4 activates the Cdc42/N-wasp pathway to promote glioblastoma invasion by altering cellular protrusions. Sci. Rep. 10 (1), 14151. 10.1038/s41598-020-70822-4 32843668 PMC7447819

[B183] Yassin N. Abouzid S. F. El-Kalaawy A. M. Ali T. M. Almehmadi M. M. Ahmed O. M. (2022). Silybum marianum total extract, silymarin and silibinin abate hepatocarcinogenesis and hepatocellular carcinoma growth via modulation of the HGF/c-Met, Wnt/β-catenin, and PI3K/Akt/mTOR signaling pathways. Biomed. Pharmacother. 145, 112409. 10.1016/j.biopha.2021.112409 34781148

[B184] Yeshurun L. Azhari H. (2016). Non-invasive measurement of thermal diffusivity using high-intensity focused ultrasound and through-transmission ultrasonic imaging. Ultrasound Med. Biol. 42 (1), 243–256. 10.1016/j.ultrasmedbio.2015.09.004 26489364

[B185] Yi L. Zhou X. Li T. Liu P. Hai L. Tong L. (2019). Notch1 signaling pathway promotes invasion, self-renewal and growth of glioma initiating cells via modulating chemokine system CXCL12/CXCR4. J. Exp. Clin. Cancer Res. 38 (1), 339. 10.1186/s13046-019-1319-4 31382985 PMC6683584

[B186] Yin J. Yin Q. Liang B. Mi R. Ai H. Chen L. (2021). Retraction Note: Chrysophanol suppresses growth and metastasis of T cell acute lymphoblastic leukemia via miR-9/PD-L1 axis. Naunyn Schmiedeb. Arch. Pharmacol. 394 (3), 571. 10.1007/s00210-020-02026-6 33247764

[B187] Yu X. Wang M. Zuo J. Wahafu A. Mao P. Li R. (2019). Nuclear factor I A promotes temozolomide resistance in glioblastoma via activation of nuclear factor κB pathway. Life Sci. 236, 116917. 10.1016/j.lfs.2019.116917 31614149

[B188] Yuksel B. Hizli D. A. Sahin F. Sahin K. Turkel N. (2023). Cannabinoid compounds in combination with curcumin and piperine display an anti-tumorigenic effect against colon cancer cells. Front. Pharmacol. 14, 1145666. 10.3389/fphar.2023.1145666 37180710 PMC10169831

[B189] Yurekli B. S. Karaca B. Kisim A. Bozkurt E. Atmaca H. Cetinkalp S. (2018). AT-101 acts as anti-proliferative and hormone suppressive agent in mouse pituitary corticotroph tumor cells. J. Endocrinol. Invest. 41 (2), 233–240. 10.1007/s40618-017-0733-8 28730425

[B190] Yusuf M. A. Singh B. N. Sudheer S. Kharwar R. N. Siddiqui S. Abdel-Azeem A. M. (2019). Chrysophanol: A natural anthraquinone with multifaceted biotherapeutic potential. Biomolecules 9 (2), 68. 10.3390/biom9020068 30781696 PMC6406798

[B191] Zhai K. Mazurakova A. Koklesova L. Kubatka P. Busselberg D. (2021). Flavonoids synergistically enhance the anti-glioblastoma effects of chemotherapeutic drugs. Biomolecules 11 (12), 1841. 10.3390/biom11121841 34944485 PMC8699565

[B192] Zhang C. Wu J. Liu W. Zheng X. Zhang W. Lee C. S. (2021a). A novel hypocrellin-based assembly for sonodynamic therapy against glioblastoma. J. Mater Chem. B 10 (1), 57–63. 10.1039/d1tb01886h 34842264

[B193] Zhang J. Wang Q. Wang Q. Guo P. Wang Y. Xing Y. (2020a). Chrysophanol exhibits anti-cancer activities in lung cancer cell through regulating ROS/HIF-1a/VEGF signaling pathway. Naunyn Schmiedeb. Arch. Pharmacol. 393 (3), 469–480. 10.1007/s00210-019-01746-8 31655854

[B194] Zhang J. Wang Q. Wang Q. Guo P. Wang Y. Xing Y. (2021b). Retraction Note to: Chrysophanol exhibits anti-cancer activities in lung cancer cell through regulating ROS/HIF-1a/VEGF signaling pathway. Naunyn Schmiedeb. Arch. Pharmacol. 394 (3), 577–578. 10.1007/s00210-020-02019-5 33242123

[B195] Zhang J. Zhao A. Jia X. Li X. Liang Y. Liu Y. (2022a). Sinomenine hydrochloride promotes TSHR-dependent redifferentiation in papillary thyroid cancer. Int. J. Mol. Sci. 23 (18), 10709. 10.3390/ijms231810709 36142613 PMC9500915

[B196] Zhang Q. Wang X. Cao S. Sun Y. He X. Jiang B. (2020b). Berberine represses human gastric cancer cell growth *in vitro* and *in vivo* by inducing cytostatic autophagy via inhibition of MAPK/mTOR/p70S6K and Akt signaling pathways. Biomed. Pharmacother. 128, 110245. 10.1016/j.biopha.2020.110245 32454290

[B197] Zhang R. Yu Q. Lu W. Shen J. Zhou D. Wang Y. (2019). Grape seed procyanidin B2 promotes the autophagy and apoptosis in colorectal cancer cells via regulating PI3K/Akt signaling pathway. Onco Targets Ther. 12, 4109–4118. 10.2147/OTT.S195615 31213831 PMC6538883

[B198] Zhang T. Liu Q. Gao W. Sehgal S. A. Wu H. (2022b). The multifaceted regulation of mitophagy by endogenous metabolites. Autophagy 18 (6), 1216–1239. 10.1080/15548627.2021.1975914 34583624 PMC9225590

[B199] Zhang Z. Shi J. Nice E. C. Huang C. Shi Z. (2021c). The multifaceted role of flavonoids in cancer therapy: Leveraging autophagy with a double-edged sword. Antioxidants (Basel) 10 (7), 1138. 10.3390/antiox10071138 34356371 PMC8301186

[B200] Zhao P. Liu Q. Wang P. Li T. Wang X. Su S. (2011). Autophagic and apoptotic response to sonodynamic therapy induced cell damage in leukemia l1210 cells *in vitro* . Cancer Biother Radiopharm. 26 (2), 209–218. 10.1089/cbr.2010.0807 21539453

[B201] Zheng X. Li W. Xu H. Liu J. Ren L. Yang Y. (2021). Sinomenine ester derivative inhibits glioblastoma by inducing mitochondria-dependent apoptosis and autophagy by PI3K/AKT/mTOR and AMPK/mTOR pathway. Acta Pharm. Sin. B 11 (11), 3465–3480. 10.1016/j.apsb.2021.05.027 34900530 PMC8642618

[B202] Zhou Y. Wang Y. Wu S. Yan Y. Hu Y. Zheng Z. (2020). Sulforaphane-cysteine inhibited migration and invasion via enhancing mitophagosome fusion to lysosome in human glioblastoma cells. Cell Death Dis. 11 (9), 819. 10.1038/s41419-020-03024-5 33004792 PMC7530759

[B203] Zimmermann K. C. Bonzon C. Green D. R. (2001). The machinery of programmed cell death. Pharmacol. Ther. 92 (1), 57–70. 10.1016/s0163-7258(01)00159-0 11750036

[B204] Zou Y. Wang Y. Xu S. Liu Y. Yin J. Lovejoy D. B. (2022). Brain Co-delivery of temozolomide and cisplatin for combinatorial glioblastoma chemotherapy. Adv. Mater 34 (33), e2203958. 10.1002/adma.202203958 35738390

